# Antiviral humoral immunity as biomarker and effector in oncolytic adenovirus TILT-123 therapy

**DOI:** 10.1136/jitc-2026-016430

**Published:** 2026-11-04

**Authors:** Victor Arias, James H Clubb, Tatiana V Kudling, Elise Jirovec, Santeri Artturi Pakola, Lyna Haybout, Dafne C A Quixabeira, Mirte van der Heijden, Nea Ojala, Susanna Grönberg-Vähä-Koskela, Sini Raatikainen, Michael J Chisamore, Katriina Jalkanen, Tuomo Alanko, Jukka Kemppainen, Maria Hellesuo, Anniina Färkkilä, Matthew Stephen Block, Tine Juul Monberg, Inge Marie Svane, Johanna Mäenpää, Suvi Sorsa, Claudia Kistler, João Manuel dos Santos, Anna Kanerva, Otto Hemminki, Victor Cervera-Carrascon, Akseli Hemminki

**Affiliations:** 1 Cancer Gene Therapy Group, Translational Immunology Research Program, University of Helsinki Faculty of Medicine, Helsinki, Finland; 2 TILT Biotherapeutics Ltd, Helsinki, Finland; 3 Helsinki University Hospital Comprehensive Cancer Center, Helsinki, Finland; 4 Merck & Co Inc, Kenilworth, New Jersey, USA; 5 Docrates Cancer Center, Helsinki, Finland; 6 Department of Clinical Physiology and Nuclear Medicine, Turku University Central Hospital, Turku, Finland; 7 Institute for Molecular Medicine Finland, University of Helsinki, Helsinki, Finland; 8 Oncology, Mayo Clinic, Rochester, Minnesota, USA; 9 Department of Oncology, National Center for Cancer Immune Therapy (CCIT DK), Copenhagen University Hospital, Herlev, Denmark; 10 Department of Obstetrics and Gynecology, Helsinki University Central Hospital, Helsinki, Finland; 11 Department of Urology, Helsinki University Hospital (HUS), Helsinki, Finland

**Keywords:** Oncolytic virus, Antibody, Humoral, Tumor Microenvironment, Biomarker

## Abstract

**Background:**

Oncolytic adenoviruses are immunotherapeutic agents that selectively replicate in malignant cells, inducing direct oncolysis, and reshaping the tumor microenvironment. While neutralizing antibodies (nAbs) are known to limit viral vector efficacy, emerging evidence suggests they may also have a role in improving clinical outcomes. This study investigates the kinetics, composition, and functional roles of nAbs, and association with overall survival and response rate in patients treated with TILT-123 (igrelimogene litadenorepvec), an Ad5/3 oncolytic adenovirus encoding tumor necrosis factor and interleukin-2.

**Methods:**

Multiple serum samples from 58 patients enrolled in three TILT-123 phase I clinical trials (NCT04695327, NCT04217473, NCT05271318) were analyzed for anti-TILT-123 nAbs using a cell-based functional neutralization assay. ELISA was used to quantify IgM, IgA, and IgG targeting TILT-123. Functional antibody signaling studies included antibody-dependent cellular cytotoxicity and antibody-dependent cellular phagocytosis (ADCP) assays. Serum proteomics, peripheral blood mononuclear cell (PBMC) profiling, and tumor immunofluorescence/RNA-seq were used to immunologically contextualize nAb induction. Survival was evaluated by Kaplan-Meier analysis, and responses by RECIST v1.1. Biomarker assessment for clinical benefit was evaluated using confusion matrices.

**Results:**

Cross-trial analysis demonstrated induction of nAb titers against TILT-123. Patients achieving high neutralizing titers early on-treatment (days 8–22) had longer survival (median OS 280 vs 190 days) and higher response rates. Baseline anti-TILT-123 IgG and IgM levels correlated with prolonged OS. ADCP activity on day 36 was associated with improved survival and disease control. Integrated serum, PBMC, and tumor IF/RNA-seq analyses supported humoral immune remodeling and increased immune trafficking and identified lymphotoxin-α as a systemic driver of early nAb induction. Early on-treatment high nAb titers emerged as the strongest single biomarker of clinical benefit, while combinatorial analyses suggested that incorporating additional factors, such as baseline nAb titers or lymphotoxin-α levels, may enhance biomarker suitability.

**Conclusions:**

High early on-treatment anti-TILT-123 nAb titers were associated with improved clinical outcomes, while ADCP activity and a pro-humoral systemic milieu supported functional roles beyond viral neutralization. These findings identify nAbs and antiviral humoral immunity as candidate biomarkers and potential effector mechanisms in oncolytic virus therapy development.

## Graphical abstract

**Graphical abstract:**
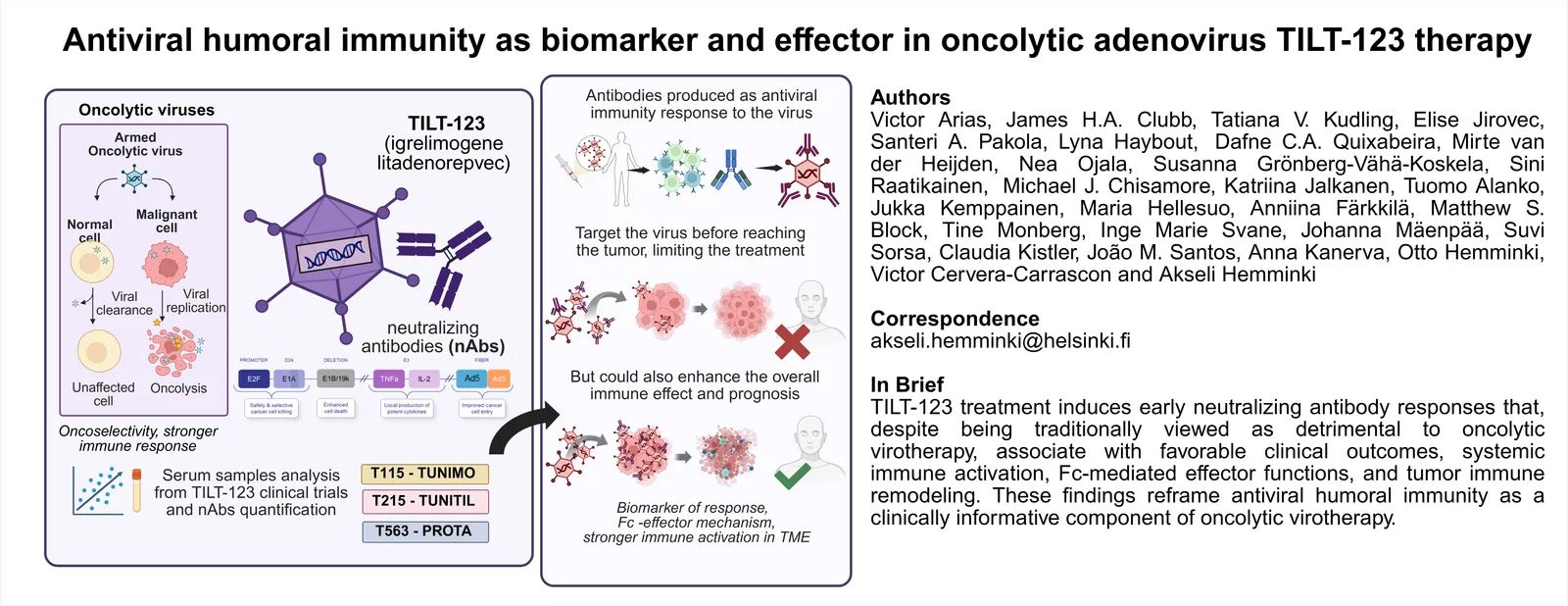

**WHAT IS ALREADY KNOWN ON THIS TOPIC**

- Oncolytic viruses induce both antitumor and antiviral immune responses. Antiviral humoral immunity, particularly neutralizing antibodies, has traditionally been viewed as a barrier to viral persistence and delivery, potentially limiting therapeutic efficacy. Whether these responses are merely restrictive or also reflect clinically meaningful immune activation remains incompletely understood.

**WHAT THIS STUDY ADDS**

- This study challenges the assumption that antiviral humoral immunity is solely detrimental in oncolytic virotherapy. In patients treated with TILT-123, early humoral responses were linked to favorable clinical outcomes and reflected broader immune activation rather than simple viral neutralization.

**HOW THIS STUDY MIGHT AFFECT RESEARCH, PRACTICE OR POLICY**

- These findings suggest that antiviral humoral responses and specifically neutralizing antibodies should not be interpreted only as unfavorable responses in oncolytic virus therapy. Instead, these responses may help identify patients who develop productive treatment-induced immunity and provide clues to the immune mechanisms associated with clinical benefit.

## Background

 Cancer remains one of the leading global health challenges and the second leading cause of death worldwide, accounting for nearly 10 million deaths in 2022.^1^ Despite advances in detection and therapy, tumor heterogeneity continues to limit durable responses, emphasizing the need for more personalized therapeutic strategies.^2 3^ Immunotherapies, particularly immune checkpoint inhibitors (ICIs), adoptive cell therapies, and cytokines, can produce long-term benefits in subsets of patients, but many tumors resist or relapse, often due to an immunosuppressive tumor microenvironment (TME) that limits effective immune infiltration.^4 5^ This motivates strategies that activate the TME while inducing systemic immunity against disseminated disease and recurrence.^6 7^

Oncolytic viruses (OVs) represent a promising strategy addressing this unmet need. They selectively infect and lyse malignant cells, enabling both direct tumor debulking and immune priming through release of tumor antigens and danger-associated molecular patterns, which stimulate innate and adaptive responses.^8 9^ In addition, OVs can be engineered to deliver therapeutic transgenes, usually immunostimulatory molecules that enhance immune activation and recruitment into the TME.^10^ The clinical approval of talimogene laherparepvec (T-VEC) for melanoma, teserpaturev (Delytact) for malignant glioma in Japan, together with the growing number of ongoing trials across multiple OV backbones, underscores the translational potential of this therapeutic approach.^11 12^

The efficacy of OV therapy, however, is closely linked to patient immune responses. While intended to promote long-term antitumor immunity, OVs also elicit antiviral responses, ranging from rapid innate mechanisms (interferon and chemokine signaling, complement system) to later adaptive responses (generation of virus-specific T and B cell responses).^12 13^ Neutralizing antibodies (nAbs) have historically been viewed as the main limitation to OV bioavailability, and this perspective has shaped the design of many OV dosing strategies, with repeated administration often considered less effective due to pre-existing or treatment-induced antiviral immunity.^12–14^ Nevertheless, emerging evidence challenges the traditional view of nAbs as purely detrimental.^15–17^ In settings where tumor infection is effective and immune activation is appropriately timed, antiviral immunity may be associated with improved outcomes and synergize with antitumor responses.^12–14^ Understanding this duality is therefore critical for optimizing OV clinical efficacy.

TILT-123 (igrelimogene litadenorepvec) is a next-generation oncolytic adenovirus engineered to express tumor necrosis factor (TNF) and interleukin 2 (IL-2). This dual-armed virus was designed to combine the direct cytolytic activity of adenoviral replication with the immune-stimulatory properties of the transgene cytokines, thereby enhancing T cell activation and intratumoral recruitment.^18–20^ TILT-123 is currently being investigated in multiple ongoing clinical trials in patients with advanced solid tumors and has suggested a potential benefit for patients having high nAbs on baseline and during treatment.^16 21–23^ Here, we build on these observations by dissecting the dynamics and functional consequences of nAb responses in patients treated with TILT-123, combining clinical outcome analyses with detailed immune and proteomic profiling to clarify their mechanistic and biomarker relevance.

## Methods

### TILT-123 clinical trials and sample collection

Patients were enrolled across three phase I TILT-123 clinical trials: T115-TUNIMO cohorts 1–7 (NCT04695327; n=26), T215-TUNITIL (NCT04217473; n=17), and T563-PROTA phase Ia (NCT05271318; n=15), comprising 58 participants in total. Trial designs, eligibility criteria, dosing schedules, and administration routes have been previously described. Patients received TILT-123 intravenously, intratumorally, and/or intraperitoneally according to the respective trial protocols. Serum, peripheral blood mononuclear cells (PBMCs), tumor biopsies, and clinical data were collected at baseline and predefined on-treatment timepoints, depending on protocol, and sample availability. Patient-level clinical characteristics, dose, route of administration, and sample availability are provided in online supplemental table 1. All patients provided informed consent before trial participation.

### Assessment of treatment response

Treatment response was assessed using RECIST version 1.1, iRECIST, and positron emission tomography (PET)-based criteria, as available: complete response (CR), partial response (PR), stable disease (SD), or progressive disease (PD), and evaluation equivalents. Overall survival (OS) and progression-free survival were retrieved from clinical trial records using a data cut-off of 01/07/25. Disease control rate (DCR) and objective response rate (ORR) were calculated per RECIST version 1.1 as DCR=(CR+PR+SD)/N and ORR=(CR+PR)/N, where N corresponds to the number of evaluable patients. For biomarker analyses, patients were classified into clinical benefit or non-clinical benefit groups based on achieving stable disease or better by RECIST version 1.1 and/or OS above the median of the corresponding trial. Patient-level response classification is provided in online supplemental table 1.

### Cell lines and viruses

Human cell line A549 (lung adenocarcinoma) was obtained from American Type Culture Collection (ATCC; LGS Standards, USA). The construction of the oncolytic adenovirus TILT-123 (Ad5/3-E2F-D24-hTNFa-IRES-hIL-2) is described in a previous publication.^18^

### NAb assay/serum anti-adenovirus antibodies

Anti-adenovirus antibodies were analyzed using a previously described nAb assay.^24^ Briefly, serial dilutions of patient sera were added onto plated A549 cells followed by the addition of replication-incompetent luciferase-expressing Ad5/3-Luc1 virus. Luciferase expression was then detected using a commercial kit (Promega, WI, USA). The dilution which blocked 80% of luciferase expression was defined as the nAb titer.

### Immunoglobulin chain isotyping

Broad anti-adenovirus IgG, IgA, and IgM levels were quantified in patient serum samples using commercial Human Anti-Adenovirus IgG, IgA, and IgM ELISA kits (Abcam, Cambridge, UK), according to the manufacturer’s instructions. Serum samples were analyzed in duplicate at a 1:100 dilution; optical density was measured at 450 nm using a Hidex Sense reader (Hidex, Turku, Finland), and results were expressed as standard units (SU).

Specific anti-TILT-123 IgG, IgA, and IgM levels were quantified using in-house ELISAs using Human IgG, Human IgA, and Human IgM Uncoated ELISA Kit (ThermoFisher, MA, USA) with purified TILT-123 as coating antigen. Briefly, 96-well plates were coated overnight with purified TILT-123 virus (1×10⁸ VP/well), blocked, and incubated with serum samples in duplicate at a 1:100 dilution. Bound immunoglobulins were detected using biotin-conjugated anti-human IgG, IgA, or IgM antibodies followed by HRP-based detection. Optical density was measured at 450 nm using a Hidex Sense reader (Hidex), and results were expressed as background-subtracted OD450 values.

### Antibody-dependent functional assays

Antibody-dependent cellular cytotoxicity (ADCC) and antibody-dependent cellular phagocytosis (ADCP) activities were measured in patient serum samples using ADCC Reporter Bioassay, V variant, and ADCP Reporter Bioassay (Promega), according to the manufacturer’s instructions. Briefly, A549 target cells were seeded in 96-well plates at a density of 20,000 cells per well and infected with TILT-123 (1000 VP per cell) for 48 hours. Serum samples were diluted 1:100 in assay-specific media and incubated with the infected target cells in triplicates together with effector cells. Luminescence was measured using a Hidex Sense reader (Hidex) after 4 hours (ADCP) and 6 hours (ADCC). Results were expressed as bridged relative luminescence units after subtraction of background signal (wells without serum). Available T563-PROTA sera were additionally tested against matched TILT-123-infected and uninfected A549 cells to assess nonspecific serum-dependent activity. Infection-specific activity was calculated after no-serum background correction and normalized using a common bridging sample.

### Serum proteomics analysis

Serum proteome (crosstrial baseline samples, T563 trial) was analyzed with Olink Target 96 ImmunoOncology panel (Olink, Uppsala, Sweden). Absolute protein concentrations provided by the manufacturer were later used for downstream analyses.

### Clinical trial PBMCs phenotyping

Cryopreserved PBMC samples were thawed, rested, Fc-blocked, and stained with antibodies against CD3, CD4, CD8, CD19, CD38, and CD56. Antibody clones, fluorochromes, vendors, and dilutions are provided in online supplemental table 2. Samples were acquired on a Novocyte Quanteon (Agilent Technologies, CA, USA) flow cytometer and analyzed using FlowJo V.10.10 (Becton, Dickinson and Company). Single-stained and unstained controls were used for compensation and gating. This analysis included available samples from patients enrolled in T115-TUNIMO and T563-PROTA; T215-TUNITIL samples were not included due to sample availability. B-cell subsets were identified using a gating strategy adapted from Clavarino *et al*.^25^ The complete gating strategy is provided in online supplemental figure 5D.

### Modulation of non-trial PBMCs by lymphotoxin-α

PBMCs from healthy donors were isolated from whole blood by density-gradient separation and seeded at 2×10⁵ cells per well. Cells were exposed to TILT-123 at 100 VP/cell in combination with recombinant human lymphotoxin-α (LTa) at 0, 1, 10, or 50 ng/mL; uninfected cells were used as controls. Cells expressing CD3, CD19, CD38, CD10, and CD56 were evaluated by flow cytometry. Antibody details are provided in online supplemental table 2. Samples were acquired on a Novocyte Quanteon (Agilent Technologies) flow cytometer and analyzed using FlowJo V.10.10 (Becton), with single-stained and unstained controls used for compensation and gating. The same core gating strategy used for the clinical-trial PBMC analysis was applied to this experiment (online supplemental figure 5D).

### IHC, multiplex immunofluorescence, and viral DNA detection

Formalin-fixed, paraffin-embedded tumor biopsies were analyzed by H&E, immunohistochemistry (IHC), and multiplex immunofluorescence (IF). Adenoviral protein was detected by IHC using an anti-adenovirus hexon antibody (Millipore, AB1056, 1:1000), while multiplex IF was used to assess immune infiltration and tumor markers, including T-cell, B-cell, NK-cell, myeloid, checkpoint, epithelial, and cytokeratin markers. Antibody details are provided in online supplemental table 2. Slides were digitally scanned and analyzed using Ilastik and CellProfiler for tissue segmentation and single-cell quantification. Samples were considered IF-positive when immune cell infiltration was detected within tumor-positive regions of interest. Viral DNA was extracted from whole blood and tumor biopsies and quantified by qPCR targeting the IRES-hIL2 sequence unique to TILT-123. Results were expressed as viral particles per mL of blood or per µg of tissue DNA. For integrative analyses, IHC, IF, and qPCR positivity were compiled into binary yes/no variables, as shown in online supplemental table 3.

### Transcriptomic analysis of tumor biopsies

RNA was extracted from available tumor biopsy samples on day 8 and 36 using the RNA Purification Mini Kit (Macherey-Nagel, Germany; Cat. 740955). Samples that failed quality control (low concentration or poor RNA integrity) were excluded from analysis. Gene expression was assessed with the NanoString nCounter PanCancer Immune Profiling Panel (NanoString Technologies, Seattle, WA, USA), supplemented with custom probes targeting adenovirus-associated genes (Ad5 hexon, Ad3 fiber, penton, and the 24-bp deleted Ad5 E1A). Hybridized samples were processed on the nCounter Digital Analyzer according to the manufacturer’s instructions. To account for batch effects, RNA expression levels were normalized using bridging samples.

### Validation of patient population

Patients treated in the Advanced Therapy Access Program (ATAP) between 2007 and 2012, with available lymphocyte count data, were included as an external validation dataset. A total of 76 patients, varying in tumor type and with available data for pretreatment and several post-treatment timepoints for nAbs titer, were used for this analysis. Patient demographics of included ATAP patients are shown in online supplemental table 4.

### Graphical illustrations and statistics

Graphs and statistics were produced with GraphPad Prism V.9.4.1. (GraphPad Software, MA, USA), RStudio V.4.3.3 (Posit Software, PBC, MA, USA), and BioRender.com.

Normality was assessed using the Shapiro-Wilk test. Pairwise comparisons were performed using the t-test, Wilcoxon, and Mann-Whitney tests. For multiple comparisons, one-way analysis of variance (with Tukey statistical correction) and Kruskal-Wallis (with Dunn statistical correction) tests were used. Correlations were performed using Pearson or Spearman methods, based on the normality of the sample distribution. Survival analyses were conducted using Kaplan-Meier estimates with log-rank (Mantel-Cox) or Gehan-Breslow-Wilcoxon testing*,* depending on the event distribution in each case. For transcriptomic analysis for differential gene expression, multiple Mann-Whitney tests were performed.

pROC package (V.1.19.0.1) from RStudio was used to generate the confusion matrices for binary classification (clinical benefit vs no benefit) and false positive/negative predictions based on accuracy, sensitivity, and specificity, in order to assess the predictive performance of candidate biomarkers. gprofiler2 package (V.0.2.4) was used to perform functional enrichment by over-representation analysis from differentially expressed genes on day 8 and 36 from the transcriptomic analysis with p<0.1. These last analysis results can be found in online supplemental tables 5–8.

## Results

### NAb titers against TILT-123 rapidly increase early on-treatment

We quantified anti-TILT-123 nAbs across three clinical trials over the treatment course: T115-TUNIMO (multiple solid tumors, monotherapy), T215-TUNITIL (melanoma, TILT-123+TILs), and T563-PROTA (ovarian, TILT-123+pembrolizumab) (figure 1A). Baseline titers were often lower and near the detection limit (not detected) but increased markedly by day 8 after the first dose (p<0.0001) and remained elevated during treatment (figure 1B). Detectable baseline titers (≥1:64) were present in 46% of patients (27/58), and 69% (40/58) reached their maximum titer early on-treatment, between days 8 and 22 (figure 1C). Baseline status was also associated with later nAb development: 74% of patients with detectable baseline nAbs (20/27) developed high titers by days 8–22, whereas 81% with non-detected baseline nAbs (25/31) remained below 1:16 384 (“low nAb titer”) (figure 1D). Baseline titers correlated with day-8 titers (p<0.0001) and maximum titer (p=0.0011), supporting the previous finding (online supplemental figure 1). We did not observe dose-dependent differences at day 8 or peak titer (figure 1E,F), noting the limited assay range. Overall, TILT-123 elicited rapid, robust nAb induction and delineated an early high-titer subgroup.

**Figure 1:**
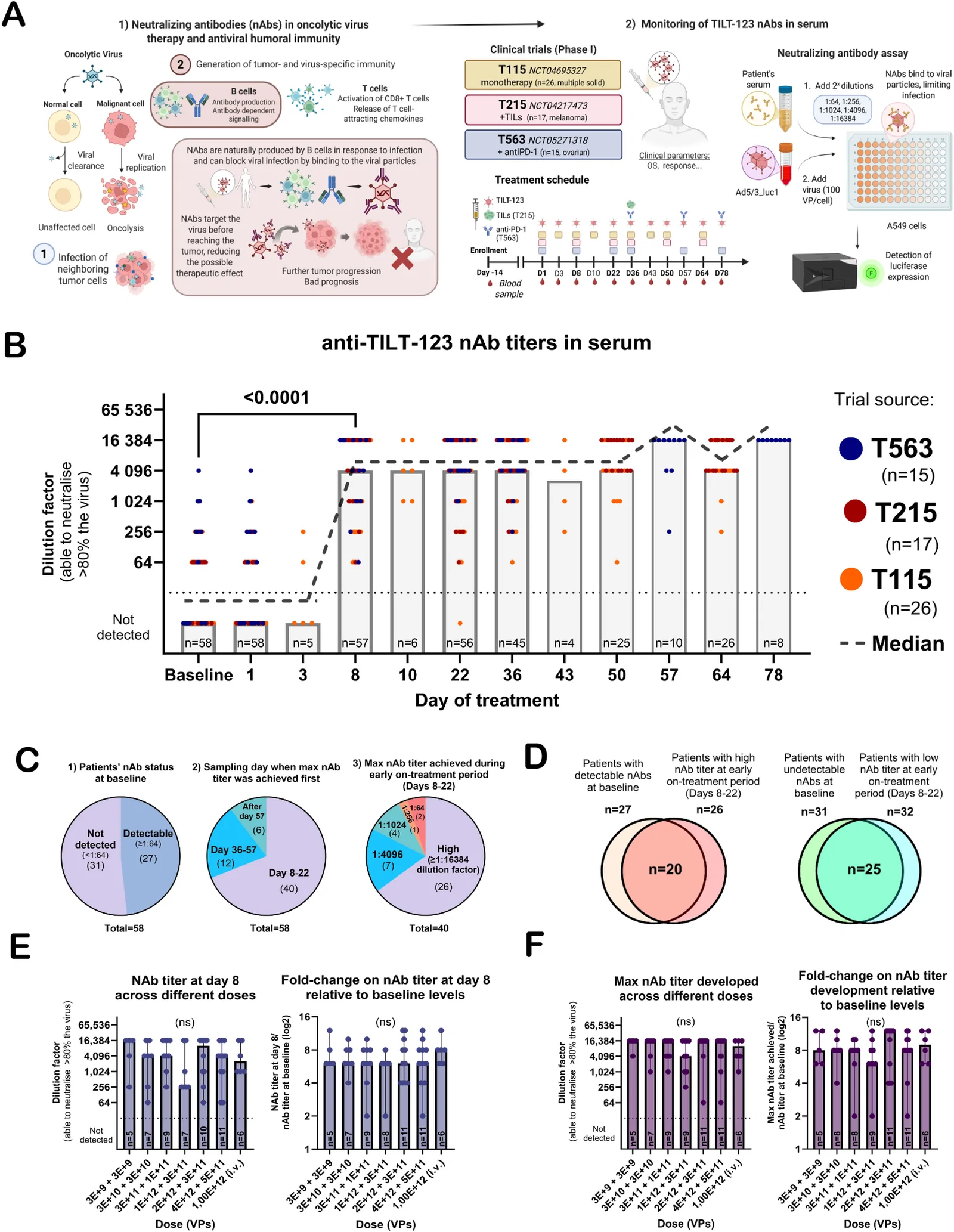
Role of neutralizing antibodies (nAbs) in oncolytic virotherapy and their characterization across TILT-123 clinical trials. (**A**) Schematic illustration of the mechanism of action of oncolytic viruses (OVs) and the historical view of nAbs as barriers to therapy, alongside a representation of the multi-trial TILT-123 study for nAbs monitoring using a functional neutralization assay. (**B**) Crosstrial monitoring of functional nAbs against Ad5/3 capsid viruses. Higher serum dilution factors indicate stronger viral blocking capacity. Each dot corresponds to one patient evaluated at the indicated timepoint, and bars indicate the median titer. Wilcoxon test was used to evaluate differences between baseline and day 8 values. (**C**) Proportion of patients with detectable baseline nAbs (≥1:64), the sampling day at which each patient reached their maximum nAb titer during treatment, and the titer achieved for those patients peaking early on-treatment (between days 8 and 22). (**D**) Overlap between patients with detectable baseline nAbs (≥1:64) and those who reached high titers (≥1:16384) early on-treatment, and between patients who had undetectable nAbs at baseline (<1:64) and those who maintained low titers early on-treatment (<1:16384). (**E**) Correlation between viral treatment dose and nAb titers measured on day 8, shown as absolute titers and as fold-change relative to baseline. (**F**) Correlation between viral treatment dose and the highest nAb titer achieved at any timepoint during treatment. Numbers on bars indicate number of patients per dose level. Kruskal-Wallis with Dunn’s multiple comparisons test was used to evaluate differences between doses. ns, not significant.

### Patients developing higher nAb titers associated with improved survival and radiological responses

We next assessed whether baseline and early on-treatment nAbs (days 8–22) could associate with OS and radiological response. Baseline titers were not associated with OS (p=0.2137), whereas day 8 titers were associated with prolonged OS (p=0.0175) (online supplemental figure 2a). Consistent with this, Kaplan-Meier analyses showed a trend for detectable baseline titers (p=0.2674, median OS=239 days) and a stronger association for achieving high titers between days 8 and 22 (p=0.0542, median OS=280 days) and at any timepoint (p=0.0396, median OS=273.5 days) (figure 2A). Trial-stratified analyses indicated that the survival association was driven by T563 and T215 (p=0.0474, median OS=306 days; p=0.0326, median OS=794 days, respectively), but not T115 (p=0.6016) (figure 2B), while baseline titers were not significant within trials (online supplemental figure 2b). Radiological response data was consistent with this: at day 8, patients with CR/PR (and immune CR/immune PR) had higher titers than those with PD (and immune confirmed progressive disease/immune unconfirmed progressive disease) by RECIST1.1 and iRECIST (both p=0.0473) (figure 2C). High titers early on-treatment associated with improved ORR/DCR by RECIST1.1 (p=0.0112, ORR 20%, DCR 65%) and iRECIST (p=0.0240, ORR 20%, DCR 60%) (figure 2D). PET responses showed a similar but weaker trend (p=0.1061), likely reflecting reduced representation of T563 (online supplemental figure 2c). Baseline titers did not associate with response across methods (online supplemental figure 2d,e). Together, these data link induction of high nAb titers early on-treatment to improved survival and radiological responses, therefore identifying them as a clinically meaningful subgroup.

**Figure 2:**
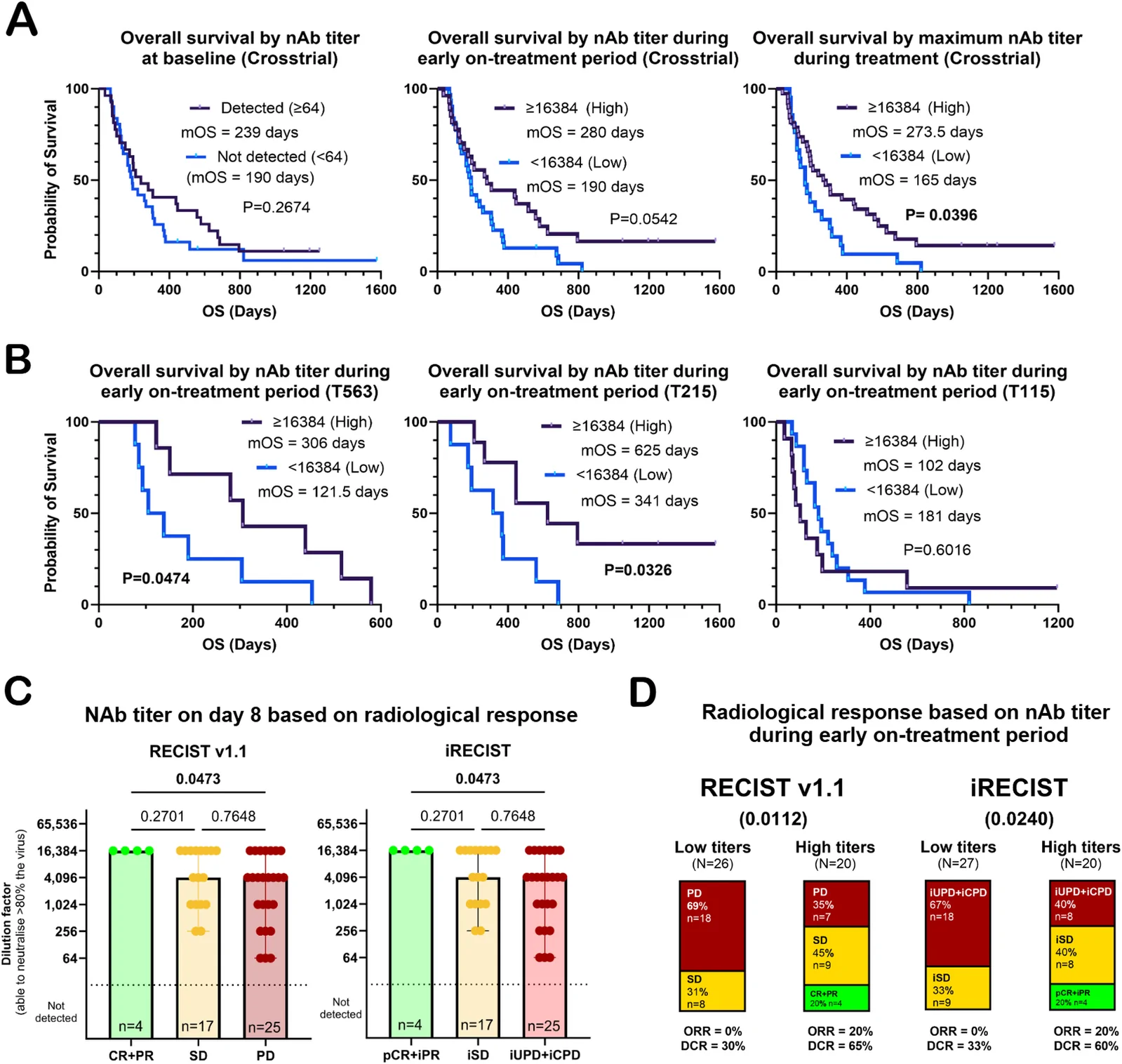
Neutralizing antibodies (nAbs) against TILT-123 correlate with longer overall survival and better response to treatment. (**A**) Crosstrial Kaplan-Meier curves for overall survival (OS) stratified by baseline nAb status (detected vs not detected using a ≥1:64 dilution cut-off), by nAb titers achieved early on-treatment (between days 8 and 22, high vs low using a ≥1:16 384 dilution cut-off), and by maximum nAb titers achieved at any timepoint (high vs low using the same cut-off). (**B**) Trial-specific Kaplan-Meier curves for OS stratified by high versus low nAb titers early on-treatment (≥1:16384). All survival analyses were evaluated using the log-rank test. (**C**) NAb titer on day 8 according to RECIST and iRECIST response categories (CR/PR/SD vs PD and equivalent). Dots correspond to each patient, and bars indicate the median value. Comparisons were evaluated using Kruskal-Wallis with Dunn’s multiple-comparisons test. (**D**) Contingency analysis for radiological response by RECIST version 1.1 and iRECIST stratified by nAb titers early on-treatment (high vs low). ORR and DCR were computed as (CR+PR)/N and (CR+PR+SD)/N, respectively, where N corresponds to the number of evaluable patients. The same definitions were applied for iRECIST-derived ORR and DCR. Fisher’s exact test was used to evaluate contingency between categories. CR, complete response; DCR, disease control rate; iCPD, immune confirmed progressive disease; iPR, immune partial response; iSD, immune stable disease; iUPD, immune unconfirmed progressive disease; mOS, median overall survival; ORR, objective response rate; OS, overall survival; pCR, pathological complete response; PD, progressive disease; PR, partial response; SD, stable disease.

### Anti–TILT-123 antibodies show isotype-specific kinetics and survival associations

Given the association between nAbs and clinical benefit, we profiled anti-TILT-123 immunoglobulin isotypes (IgM, IgA, and IgG), given their distinct roles during viral infection and their different signaling properties (online supplemental figure 3a). Anti-TILT-123 immunoglobulins were quantified by an in-house ELISA protocol using virus-coated plates (figure 3A). Across trials, isotypes showed distinct kinetics: IgM rose transiently at day 8 and declined by day 36, IgA increased at day 8 and remained elevated, and IgG, which was already detectable at baseline, showed the most pronounced induction after day 8 (figure 3A,B). Trial-stratified analyses indicated heterogeneity, with the strongest induction in T563, intermediate responses in T215, and weaker responses in T115 (figure 3B). These isotype levels per timepoint were also associated with matched patients’ OS. In cross-trial analyses, higher baseline IgG (p=0.0039) and IgM (p=0.1130), and day-36 IgG (p=0.0769), associated with prolonged OS. Within T563, baseline IgG (p=0.0582) and day-8 IgA (p=0.0415) associated with longer OS (figure 3C). Kaplan-Meier analyses confirmed improved survival in patients with higher baseline IgG (p=0.0449, median OS=304 days) or IgM (p=0.0845, median OS=304 days), with the greatest benefit in patients with simultaneously high baseline IgG and IgM (p=0.0062, median OS=316 days) (figure 3D). Notably, commercial ELISAs capturing broader anti-adenovirus antibodies similarly profiled the isotype kinetics but did not significantly associate with OS (online supplemental figure 3b–d), suggesting that the survival improvement is linked to antibodies directed against TILT-123 rather than general anti-adenovirus seroreactivity. Collectively, these data identify baseline IgG and IgM as survival-associated features within the antiviral humoral response to TILT-123.

**Figure 3:**
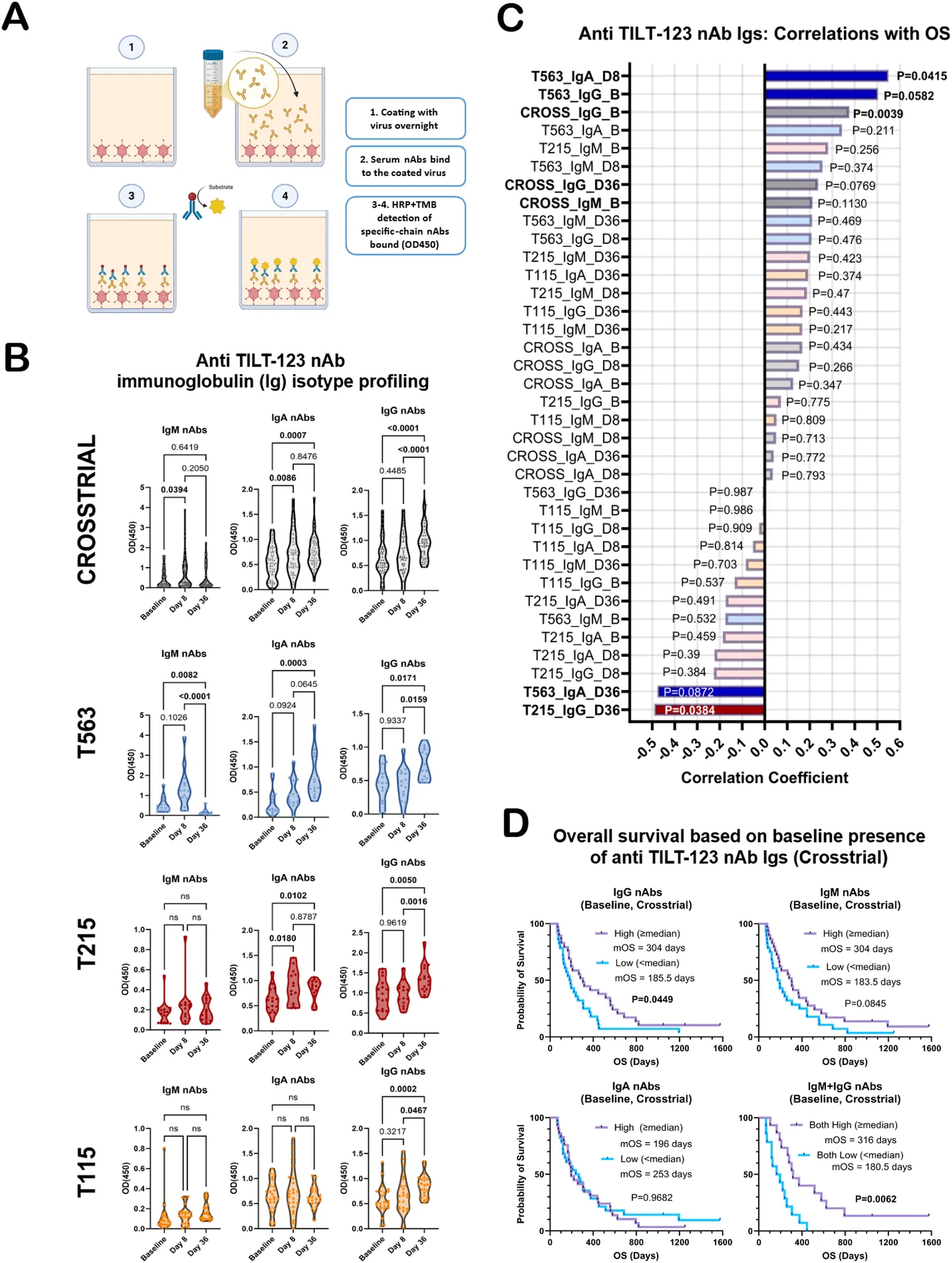
Evaluation of pre-existing and treatment-induced immunoglobulin isotypes against Ad5/3 chimeric capsid viruses. (**A**) Schematic of the assay used to detect anti-TILT123 IgM, IgA, and IgG using virus-coated plates. (**B**) Quantification of circulating anti-TILT123 IgM, IgA, and IgG levels in serum at baseline, day 8, and day 36 across all TILT-123 clinical trials (cross-trial and per-trial results). Dots correspond to each patient, and comparisons between days were performed using one-way analysis of variance. (**C**) Waterfall plots displaying correlations between each Ig isotype at each timepoint and the corresponding patient OS, shown per trial and cross-trial. Bar length indicates the correlation coefficient (Pearson/Spearman), and p values are shown next to each bar. (**D**) Cross-trial Kaplan-Meier survival curves stratified by median baseline abundance of anti-TILT123 IgG, IgM, and IgA individually, and by combined high baseline IgG+IgM. Survival was evaluated using Gehan-Breslow-Wilcoxon test. nAbs, neutralizing antibodies; ns, not significant; mOS, median overall survival; OS, overall survival.

### Antibody-driven activation of cellular phagocytosis correlates with improved clinical outcomes

To test whether anti-TILT-123 antibodies engage Fc-effector function, we quantified ADCC (NK cell-linked) and ADCP (macrophage/APC-linked) using reporter assays (figure 4A,B). Both activities were detectable at baseline, and ADCC increased by day 8 (p=0.0007) and further by day 36 (p<0.0001), whereas ADCP increased more gradually and was significantly elevated by day 36 (p<0.0001) (figure 4C,D). Day-36 ADCC and ADCP associated with prolonged OS (p=0.0291 and p=0.0046, respectively) (figure 4E), with trial heterogeneity driven largely by T563, in which day-36 ADCP showed the strongest association (p=0.003) (figure 4E). In contrast, baseline ADCC associated negatively with OS in T115 (p=0.036), while T215 showed only a trend for day-36 ADCC (p=0.1298) (online supplemental figure 4a). Consistent with the nAb-defined subgroup, ADCP (but not ADCC) at day 36 was higher in patients with high nAb titers early on-treatment (p=0.0190), particularly in T563 (p=0.0350) (figure 4F, online supplemental figure 4b). Kaplan-Meier analysis further supported this with improved survival with higher day-36 ADCP (p=0.0084, median OS=344 days) (figure 4G), and day-36 ADCP activity was also higher in patients with disease control (CR/PR/SD) versus PD by both RECIST1.1 and iRECIST (p=0.0409 for both) (figure 4H). To exclude nonspecific serum-dependent activity against A549 cells, available T563 sera were additionally tested using matched TILT-123-infected and uninfected target cells. Activity against uninfected cells was comparatively limited, and after correction for uninfected-cell activity, day-36 ADCP remained positively correlated with OS (r=0.75, p=0.003) (online supplemental figure 4c–f). In paired tumor biopsies, CD16^+^ cell frequency at day 36 correlated with OS (p=0.0491) (figure 4I), and mIF demonstrated co-localization of CD16^+^ and CD11c^+^ cells within tumor regions (figure 4J, online supplemental figure 4g). Collectively, these results indicate that anti-TILT-123 antibodies engage Fc-dependent effector pathways, with ADCP showing the most consistent association with clinical benefit.

**Figure 4:**
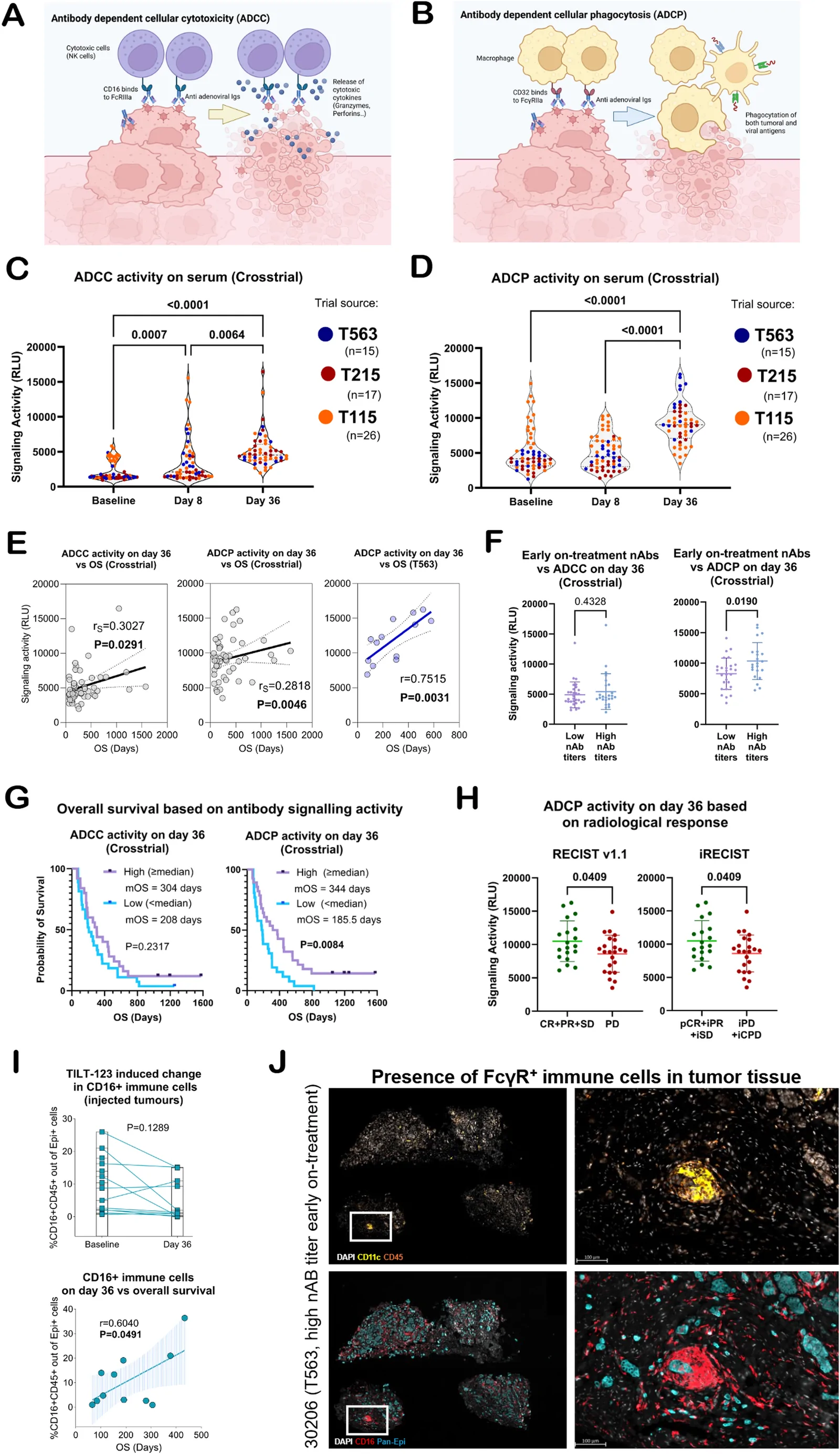
Antibody-dependent effector functions during TILT-123 treatment and their association with clinical outcome. (**A and B**) Schematic representation of (**A**) antibody-dependent cellular cytotoxicity (ADCC) and (**B**) antibody-dependent cellular phagocytosis (ADCP), illustrating Fc receptor-mediated immune activation triggered by anti-adenoviral antibodies. (**C–D**) Cross-trial quantification of (**C**) ADCC and (**D**) ADCP signaling activity at baseline, day 8, and day 36 of treatment. Each point represents an individual patient’s effector function measured from serum at the indicated timepoints. Comparisons between days were performed using one-way analysis of variance. (**E**) Correlations between ADCC and ADCP activity on day 36 and overall survival (OS) in the cross-trial cohort and the T563 trial. Correlations were performed using Pearson/Spearman test. (**F**) Pairwise comparison of ADCC and ADCP activity at day 36 between cross-trial patients stratified by high versus low early on-treatment nAb titers. Comparisons were performed using Mann-Whitney test. Dots represent individual patients, bars indicate mean±SEM. (**G**) Cross-trial Kaplan-Meier survival curves stratified by median ADCC and ADCP activity at day 36, evaluated by log-rank test. (**H**) ADCP activity on day 36 stratified by radiological response (RECIST version 1.1, iRECIST). Comparisons were performed using Mann-Whitney test. Dots represent individual patients, bars indicate mean±SEM. (**I**) Multiplex immunofluorescence (mIF)-based quantification of CD16^+^ immune cells in injected tumors at baseline and day 36 for patients with available biopsies, and correlation between CD16^+^ cell abundance and OS for day 36 samples. Pairwise comparison was performed using paired t-test, and correlation was evaluated using Pearson test. (**J**) Representative mIF images from a non-injected tumor biopsy from TILT-123-treated patient 30206, belonging to the high-nAb group. Overview images (left) show the distribution of CD11c^+^ and CD16^+^ immune cells across the tumor tissue, with boxed regions shown at higher magnification (right). The magnified images illustrate the presence of CD11c^+^ immune-cell infiltrates and accumulation of CD16^+^ cells in close proximity to epithelial tumor areas within the same tumor region. Markers are shown as follows: DAPI nuclear stain (white), CD11c (yellow), CD45 (orange), CD16 (red), and pan-epithelial marker (cyan). Scale bars, 100 µm. CR, complete response; iCPD, immune confirmed progressive disease; iPR, immune partial response; iSD, immune stable disease; iUPD, immune unconfirmed progressive disease; mOS, median overall survival; nAbs, neutralizing antibodies; OS, overall survival; pCR, pathological complete response; PD, progressive disease; PR, partial response; SD, stable disease.

### LTa shapes B-cell derived antibody responses and is associated with clinical benefit

To identify systemic correlates of early high nAb induction that may be clinically tractable, we integrated serum proteomics with PBMC profiling. Baseline proteomic screening stratified by early nAb groups in T563 nominated LTa (former TNF-β) as a candidate marker (figure 5A). Baseline LTa was higher in patients who later developed high nAb titers, associated with OS (p=0.0312), and showed a trend with disease control (PR/SD vs PD, p=0.0954) (figure 5B). In T563, LTa remained higher across timepoints in the high-titer group, peaking at day 1 before declining (figure 5C), preceding the nAb peak at day 8 (figure 5D). Extending analyses across trials supported an association between elevated baseline LTa and longer OS, strongest in the pooled cohort (p=0.0293, median OS=306 days), with similar trends in T563 (p=0.0960, median OS=293 days), and T215 (p=0.1396, median OS=686 days), but not T115 (figure 5E, online supplemental figure 5a). Given reported roles for LTa in TNF-superfamily signaling and B-cell differentiation (figure 5F),^26 27^ we examined PBMC dynamics seeking overall treatment-associated changes: plasma B cells (p<0.0001), B cells (p=0.0120), and NK cells (p=0.0230) increased from baseline to day 8 (figure 5G), and these changes correlated with nAb fold-changes and thus magnitude of the nAb response, although not with LTa levels (figure 5H). In vitro stimulation of healthy donor PBMCs supported mechanistic plausibility, with LTa promoting plasma B-cell proliferation in a dose-dependent manner in the absence and presence of TILT-123 (p=0.0435 and p=0.0666) and a similar trend for NK cells with virus (p=0.0613) (online supplemental figure 5c). Together, these data link baseline LTa to early on-treatment high nAb induction and survival and suggest that LTa can modulate cellular programs relevant to antiviral humoral immunity.

**Figure 5:**
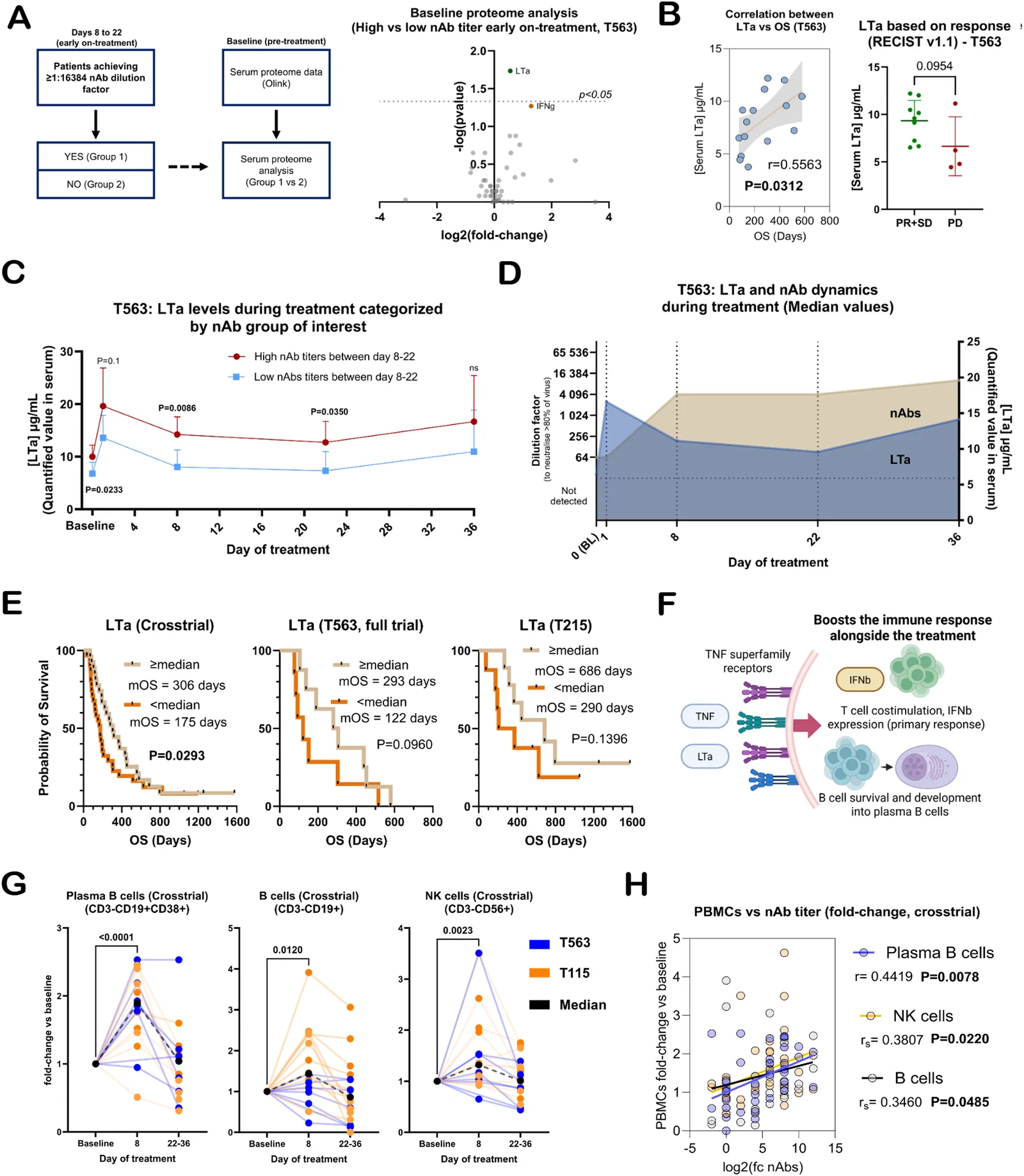
Serum and blood immune environment analysis identifies lymphotoxin-α (LTa) as a key inducer of neutralizing antibodies (nAbs) and B-cell-mediated immunity. (**A**) Analysis pipeline applied to baseline serum samples stratified by early on-treatment nAb titer, and volcano plot showing differentially expressed proteins. (**B**) Pearson correlation between baseline LTa levels and OS, and baseline LTa levels stratified by RECIST response in the T563 trial, evaluated by unpaired t-test. Dots represent individual patients, bars indicate mean±SEM. (**C**) Longitudinal analysis of LTa levels during treatment (up to day 36) in T563, stratified by high versus low post-treatment nAb titer groups. Comparisons at each timepoint were performed using unpaired t-test, bars indicate mean±SEM. (**D**) Temporal comparison of LTa and nAb dynamics throughout the T563 treatment schedule, suggesting a potential feedback relationship. (**E**) Kaplan-Meier survival curves stratified by baseline LTa abundance in serum (crosstrial, and for T563 and T215), evaluated by Gehan-Breslow-Wilcoxon test. (**F**) Schematic representation of LTa’s immunological functions, including its role in B-cell activation and primary responses. (**G**) PBMC profiling in a subset of crosstrial patients showing fold-change in immune cell populations from baseline to day 8 and day 22/36: plasma B cells (CD3⁻CD19^+^CD38^+^), total B cells (CD3⁻CD19^+^), and NK cells (CD3⁻CD56^+^). Comparisons between days were evaluated using Wilcoxon test. (**H**) Correlation analysis between PBMC population changes and corresponding nAb titers at day 8 and day 22/36 relative to baseline. Correlations were performed using Spearman test. mOS, median overall survival; ns, not significant; OS, overall survival; PBMC, peripheral blood mononuclear cell; PD, progressive disease; PR, partial response; SD, stable disease; TNF, tumor necrosis factor.

### Early post-treatment high nAb titers and LTa may serve as potential predictive biomarkers

To evaluate clinical utility, we assessed whether early nAb induction and baseline LTa identify patients most likely to benefit from TILT-123 therapy. Across pooled and trial-specific analyses, patients with high nAb titers early on-treatment, elevated baseline LTa, or higher day-36 ADCP activity showed increased DCR, ORR, or both relative to the corresponding trial-level values (figure 6A). To integrate radiological outcome with survival in a clinically interpretable framework, we classified patients as clinical benefit (CBG: CR/PR/SD and/or OS above the trial median) versus non-benefit (non-CBG: PD and OS below the trial median). Confusion-matrix analyses showed that high nAb titers early on-treatment provided the strongest predictive performance across the full dataset and within T563 and T215 (figure 6B). T115 was not analyzed separately because early nAb titers had not shown a significant association with clinical outcome or predictive value in the preceding analyses. Predictive performance further improved when baseline LTa was combined with pretreatment and early on-treatment nAb features, including perfect classification in T215 (figure 6C), supporting a multi-parameter biomarker approach.

**Figure 6:**
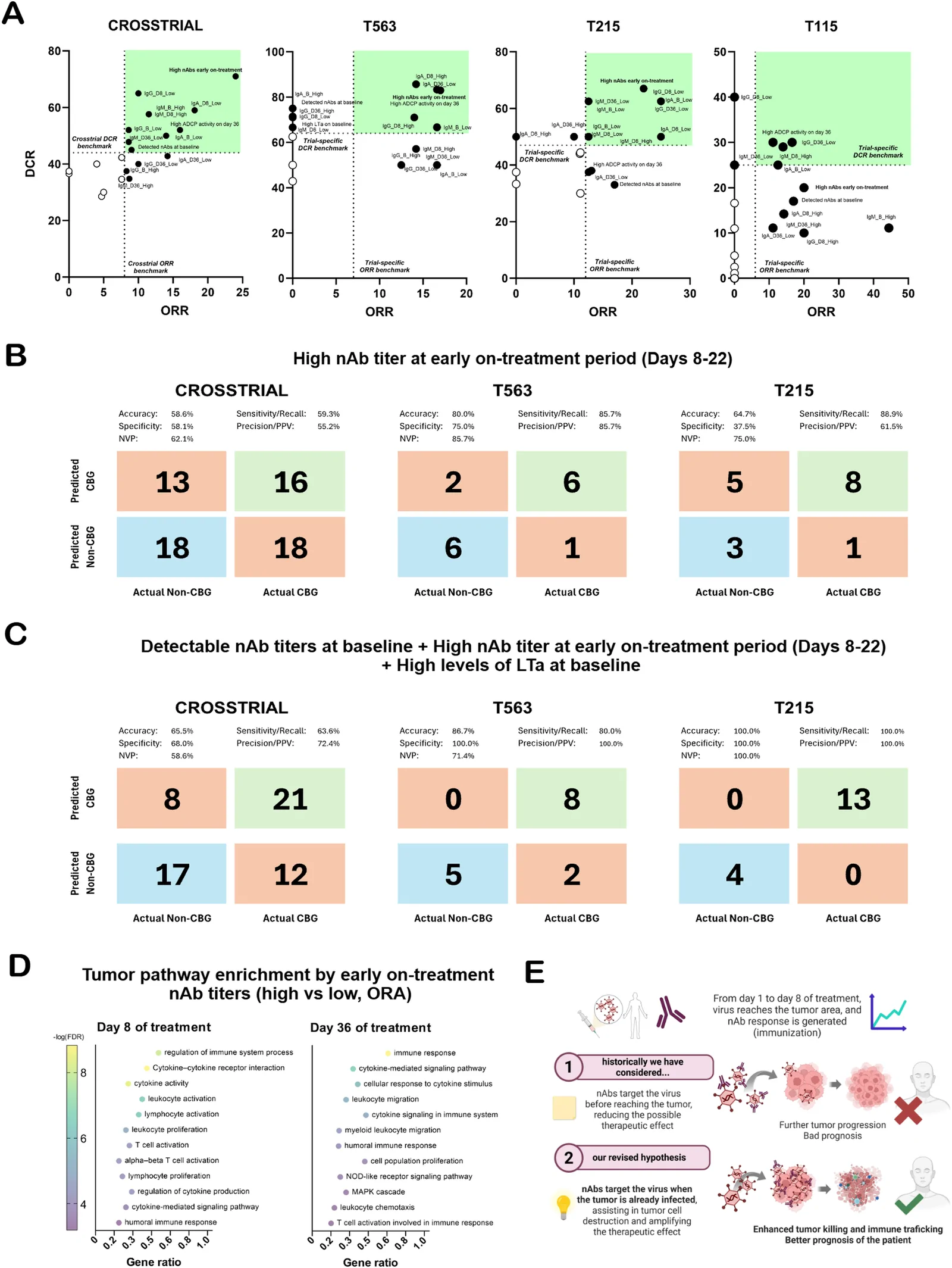
Neutralizing antibody (nAb) and lymphotoxin-α (LTa) signatures predict clinical benefit and associate with enhanced tumor immune activation in TILT-123 therapy. (**A**) Volcano plots displaying associations between candidate biomarkers and objective response rate (ORR) and disease control rate (DCR) as defined by RECIST. Dashed lines indicate trial-specific ORR and DCR benchmarks; dots represent top-ranked biomarkers identified across the study. (**B,C**) Confusion matrices showing predictive performance for classifying patients into a clinical benefit group (CBG: CR+PR+SD+longOS) versus non-CBG (PD+shortOS) in the crosstrial analysis, T563 and T215: (**B**) based on high nAb titers achieved early on-treatment (between days 8 and 22); (**C**) based on a combined model incorporating detectable baseline nAb titers, high early on-treatment nAb titers, and elevated baseline LTa levels. Box numbers correspond to predicted versus observed CBG/non-CBG classification, with summary performance metrics (accuracy, specificity, etc) shown for each scenario. (**D**) Over-representation analysis (ORA) of tumor transcriptomics from day 8 and day 36 biopsies, comparing patients with high versus low early on-treatment nAb titers. (**E**) Conceptual illustration proposing a revised model for the beneficial role of neutralizing antibodies observed in TILT-123 clinical trials. ADCP, antibody-dependent cellular phagocytosis; CR, complete response; FDR, false discovery rate; NPV, negative predictive value; OS, overall survival; PPV, positive predictive value; PR, partial response; SD, stable disease.

To link circulating markers with intratumoral biology, we compared pathway enrichment based on tumor biopsy transcriptomics from patients with early high versus low nAb titers at days 8 and 36 (figure 6D). On day 8, high nAb titers were associated with enrichment of immune regulatory and cytokine programs, including cytokine signaling/production and T- and B-cell activation and proliferation. By day 36, these patients showed persistent immune and cytokine signaling together with enrichment of leukocyte and myeloid migration/chemotaxis and NOD-like receptor and MAPK signaling, consistent with an inflamed microenvironment compatible with Fc-dependent effector activity. Together, these analyses support coupling between early nAb induction, intratumoral immune activation, and improved tumor control (figure 6E).

Finally, exploratory analyses suggested that nAb-associated effects may depend on route of administration and tumor indication. Within T115, the fully intravenously subset showed a trend toward an opposite association, with lower early on-treatment nAb titers linked to longer survival relative to the intratumorally subset (online supplemental figure 6a). In comparisons between the intravenously cohort and patients receiving equivalent doses locally (intratumorally/intraperitoneally), detectable titers at baseline (p=0.0960) or day 8 (p=0.0819) associated with poorer survival (online supplemental figure 6b). Consistent with reduced intratumoral delivery in this setting, tumor IF positivity was absent among evaluated intravenously treated patients with high nAbs, and IF/viral positivity increased after excluding the intravenously cohort (online supplemental figure 6c,d). In historical ATAP data using similar adenoviral backbones, nAb kinetics resembled those observed here, and clustering by tumor immunogenicity suggested indication-specific differences in nAb–outcome relationships (online supplemental figure 7). Collectively, these exploratory findings suggest that both biomarker performance and the direction of nAb associations may be conditioned by delivery route and tumor context.

## Discussion

In recent decades, OVs have emerged as a relevant focus in cancer immunotherapy, as they can selectively target tumors while also reshaping the immune landscape.^8^ By combining direct tumor lysis with the induction of systemic antitumor immunity, OVs act as both cytotoxic agents and in situ cancer vaccines, also creating opportunities for potential synergy with ICIs and other treatments.^9 28^ However, their efficacy, and in part the challenge of broad clinical approval, has been strongly influenced by the magnitude of the patient antiviral response, which can restrict viral bioavailability and reduce therapeutic efficacy.^29 30^ NAbs have therefore been viewed as key barriers, rapidly inactivating circulating virions, and thus, many strategies have focused on bypassing them.^14 31^

Nevertheless, over the last few years, antiviral immunity is being recognized as a potential ally in OV therapy, challenging the assumption that pre-existing or treatment-induced immunity necessarily limits efficacy.^14^ Recent findings have highlighted that these responses may support therapy by enhancing immune infiltration and antigen presentation, shifting attention toward how viral immunodominance can be harnessed to promote durable antitumor immunity.^13 14 32^ Preclinical studies on HSV-1 found that pre-existing immunity can enhance tumor growth control and T cell function.^15^ Clinical hAd5 studies suggest that humoral immunity is more complex than neutralization alone: induced nAbs did not fully block infection, and human serum contained infection-promoting activity.^17^ However, major clinical OV platforms such as T-VEC and ONCOS-102, despite inducing robust antiviral antibodies, have not shown clear clinical benefit linked to nAb induction.^33 34^ To date, only the TILT-123 PROTA (T563) clinical trial has suggested a survival association with anti-TILT-123 nAbs, motivating the analyses presented here.^16^

We therefore profiled antiviral humoral immunity, in this case starting with nAbs, in TILT-123-treated patients. Higher nAb titers induced early-on treatment were associated with improved clinical outcomes, supporting nAbs as candidate biomarkers of therapeutic activity. Associations extended beyond titers to include isotype kinetics, Fc-effector activity, and circulating LTa, indicating a multifactorial humoral signature linked to benefit that may be combined for prediction. Interestingly, in a subset of patients, nAb titers remained elevated after treatment discontinuation, consistent with sustained antigen exposure or long-lived plasma cell responses, suggesting persistent immune engagement beyond dosing.^35^

Isotype profiling provided mechanistic context for these associations. Anti-TILT-123 IgM, IgA, and IgG displayed expected kinetics and distinct prognostic implications that, consistent with the broader nAb findings, varied across trials.^36^ Baseline IgM and IgG were associated with improved outcomes, while IgG showed the most consistent association with survival. The presence of TILT-123-binding IgG at baseline despite undetectable neutralizing activity in some patients likely reflects the distinction between antibody binding and functional neutralization. ELISA detects both neutralizing and non-neutralizing virus-binding antibodies, whereas the nAb assay requires sufficient concentrations of antibodies targeting functionally relevant epitopes to inhibit viral infection. Patients with both elevated baseline IgM and IgG had the best outcomes, suggesting complementary contributions of early and established humoral responses.^37 38^ Notably, IgM-mediated antibody-dependent enhancement of viral entry has been observed in other TILT-123 studies.^39^ Functional assays further supported activity beyond neutralization: ADCC and ADCP were associated with longer survival, with ADCP showing the strongest association and tracking with disease control. This is likely due to its role in facilitating uptake and presentation of viral, but also indirectly tumor antigens.^40^ Thus, these findings suggest that ADCP may bridge antiviral and antitumor immunity, converting humoral responses into drivers of long-term clinical benefit. Collectively, these data implicate IgM/IgG-driven Fc-effector functions as a key mechanism by which nAbs could exert their clinical benefits.

Tumor-level analyses further aligned with this Fc-effector interpretation. mIF showed frequent co-localization of CD16^+^ cells with CD11c^+^ macrophages/antigen-presenting cells, consistent with a spatially coordinated Fc-effector activity in situ.^41^ Likewise, transcriptomic pathway enrichment of day-8 and day-36 biopsies comparing patients with high versus low nAb titers revealed cytokine/chemokine, leukocyte migration, and immune activation signatures, consistent with increased immune trafficking in the TME. Although these tumor analyses were correlative and limited by paired sample availability, this convergence of serum isotype and Fc-function readouts with histological and transcriptomic signals supports a model in which nAbs help orchestrate Fc-dependent effector mechanisms impacting immune infiltration in the TME, thereby contributing to the clinical activity of TILT-123.

Beyond antibody readouts, LTa emerged as a systemic correlate of nAb induction. Elevated baseline LTa associated with high early on-treatment nAb titers and with longer survival, consistent with reported roles in B-cell survival, plasma cell differentiation, and antiviral cytokine signaling.^42^ Although LTa did not correlate with PBMC B-cell subsets (likely reflecting sampling bias from the T115 trial, where nAbs were less predictive), its association with nAbs was robust. In vitro experiments with naïve PBMCs from healthy donors supported mechanistic plausibility, showing that LTa could promote plasma cell differentiation. Together, these data highlight that cytokine networks can condition antiviral humoral immunity in OV therapy and raise the possibility for the LTa axis as a biomarker and potential modulator in future studies.

NAb-associated benefit was trial dependent in both magnitude and direction, with a detrimental association in T115 (multiple solid tumors) and more favorable patterns in T563 (ovarian) and T215 (melanoma). These data support a context-dependent model in which the clinical relevance of nAbs may vary with tumor indication and intrinsic immunogenicity. This premise was consistent with exploratory clustering of historical nAb data from ATAP using the same adenoviral backbone. A plausible interpretation is that tumor immunogenicity may not determine nAb induction itself, but rather the downstream immune activation and trafficking that antiviral humoral responses can accompany, which may be more effective in immunogenic settings.^43^ Nonetheless, other contributors, including tumor heterogeneity and patient clinical status, are likely to shape these inter-trial differences.^44^ Importantly, most associations were evident by day 36, before introduction of combination agents, and early on-treatment nAb kinetics were comparable across trials, suggesting that differences in early nAb induction are unlikely to be driven by trial-specific combination regimens. However, later combinations may still modulate the translation of early antiviral responses into long-term outcomes. In this regard, high nAb titers may also represent a marker of a broader, coordinated immune response to TILT-123 rather than acting as the sole therapeutic effector. Such immune activation could facilitate the activity of administered TILs in T215 or endogenous antitumor T cells enabled by PD-1 blockade in T563, potentially contributing to the outcome observed. Nevertheless, the association between ADCP activity and survival supports the possibility that antiviral antibodies may also contribute directly through Fc-mediated effector mechanisms. Thus, humoral immunity may function both as a biomarker of effective immune engagement and as one component of a broader cellular and humoral antitumor response.

Finally, route of administration may further condition the impact of nAbs. Patients who received local TILT-123 delivery (intratumorally/intraperitoneally) after an initial intravenously phase tended to show improved outcomes with early high nAb titers, whereas in the fully intravenously cohort higher titers associated with shorter survival. These data are consistent with a two-step model in which systemic exposure primes antiviral antibodies, while subsequent local delivery enables antibody engagement within the TME once infection is established, potentially supporting Fc-effector activity and immune priming. This interpretation aligns with the experimental work by Niemann *et al*, where adenovirus-specific antibodies were retargeted to tumors using bispecific adapters to elicit NK- and T-cell-mediated antitumor activity.^45^ However, these route-based analyses are exploratory and require validation in larger, dedicated intravenously delivery cohorts.

Several limitations should be acknowledged. First, trial and subgroup-level sample sizes were modest, particularly for the fully intravenously cohort, and clinical heterogeneity and a low number of objective responders limits precision for response-based estimates. Second, analyses are correlative and do not establish causality between nAb dynamics, LTa, Fc-effector activity, and the corresponding outcomes; while ex vivo assays and tumor profiling support mechanistic plausibility, in vivo validation is needed. Third, tumor transcriptomic and PBMC analyses were available for a limited number of paired samples and should be interpreted as exploratory. Larger, more homogeneous studies with standardized dosing/sampling and targeted mechanistic experiments (as perturbing B cells/antibodies or Fc receptors) will be important to test antibody-mediated pathways to therapeutic benefit.

## Conclusions

In conclusion, our data indicate that anti-TILT-123 nAbs are not uniformly detrimental: once intratumoral infection is established, higher nAbs titers track with clinical benefit and with Fc-effector activity. Integrated isotype, functional, and tumor-level analyses support a model in which antiviral humoral immunity extends beyond neutralization and is linked to Fc-dependent effector programs and immune trafficking within the TME. High early on-treatment nAb titers and baseline LTa emerged as candidate biomarkers for patient stratification, and motivating prospective validation and assessment of the LTa axis as a potential modulator of humoral responses. These findings refine how antiviral immunity is interpreted in OV therapy and provide a rationale for incorporating humoral biomarkers into future OV and other immunotherapies’ clinical development.

## Supplementary material

10.1136/jitc-2026-016430 online supplemental file 1

10.1136/jitc-2026-016430 online supplemental file 2

10.1136/jitc-2026-016430 online supplemental file 3

## Data Availability

Data are available upon reasonable request.
