# Supplementary material for: Antiviral humoral immunity as biomarker and effector in oncolytic adenovirus TILT-123 therapy
Source: J Immunother Cancer. 2026 Nov 4;14(11):e016430. doi: 10.1136/jitc-2026-016430 (PMC13735263; doi:10.1136/jitc-2026-016430)
Supplement: Supplementary file 1 [file jitc-14-11-s002.pdf]

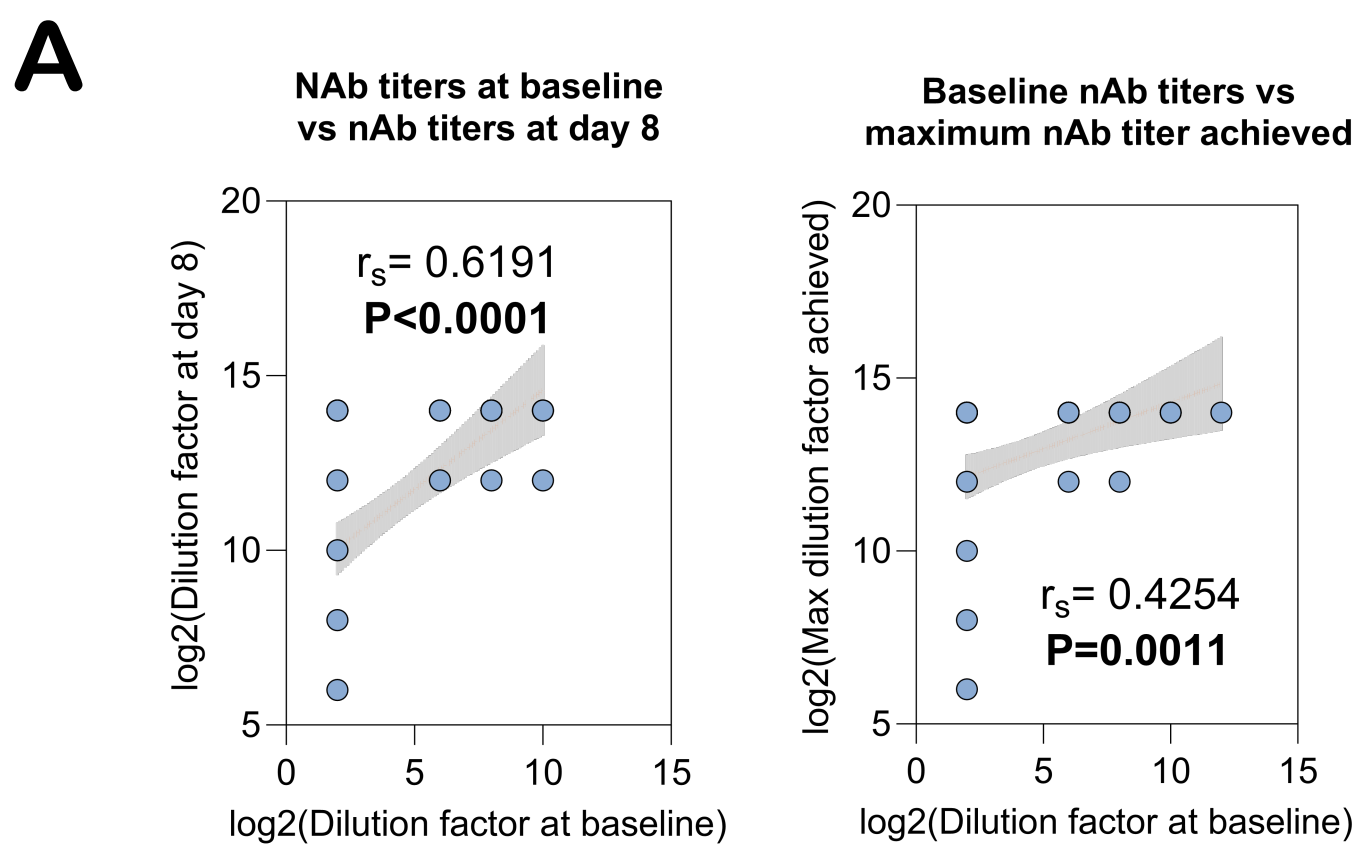

Supplementary Figure 1: Additional correlations between baseline and on-treatment neutralizing antibody titers. Correlations between baseline nAb titers and either nAb titers at day 8 or the maximum nAb titer achieved at any time during TILT-123 treatment. Correlations were performed using Spearman test.

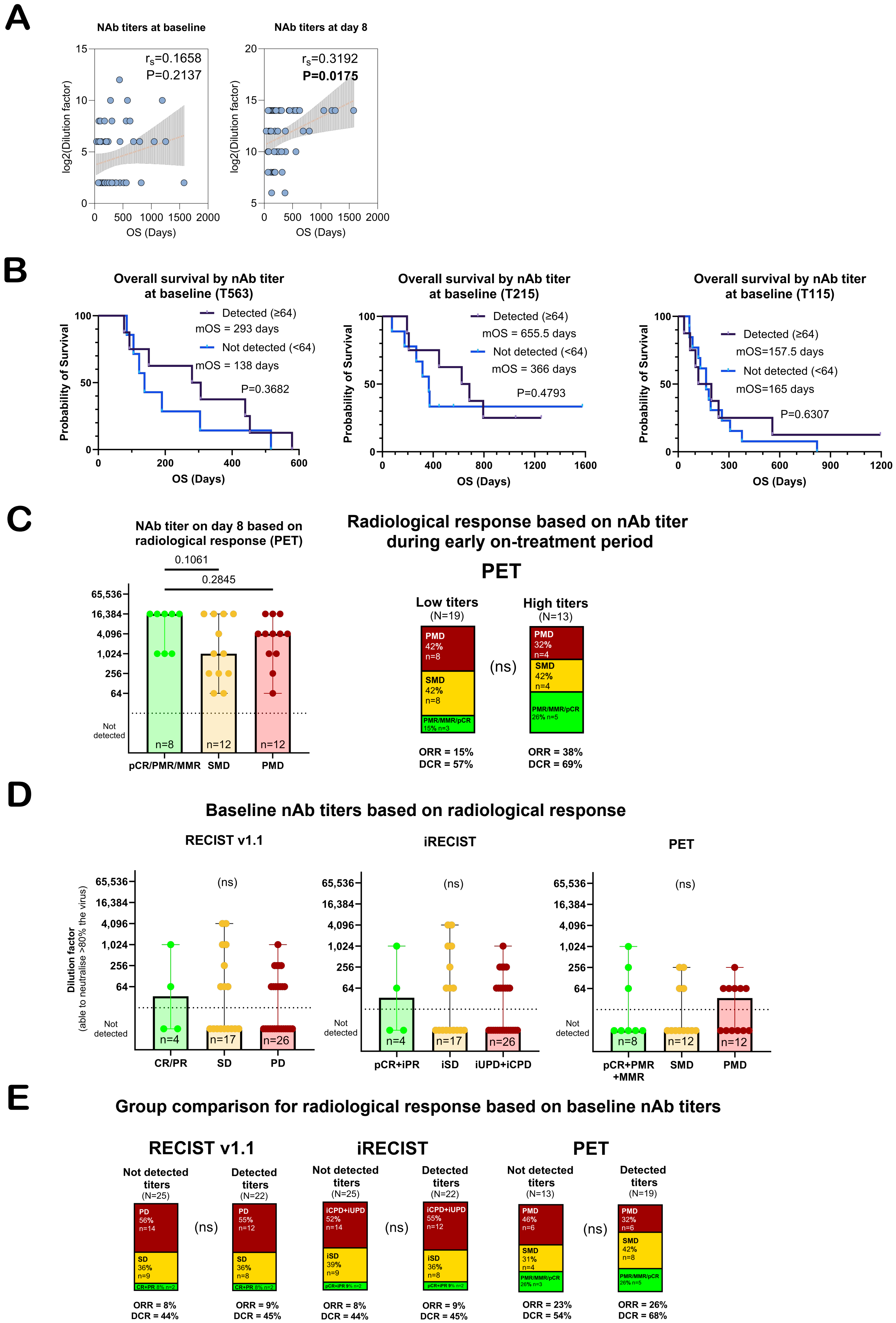

Supplementary Figure 2: Survival and radiological response based on neutralizing antibody titers across TILT-123 clinical trials. (A) Correlation analyses between nAb titers at baseline or day 8 and overall survival (OS) in patients treated with TILT-123. Correlations were performed using Spearman test. (B) Kaplan-Meier curves for OS stratified by positive versus negative baseline nAb titers for each trial (T563, T215, T115). Survival was evaluated using log-rank test. (C) nAb titers at day 8 according to PET response categories, including contingency analyses. (D) Baseline nAb titers according to RECIST v1.1, iRECIST and PET criteria. Comparisons were evaluated using Kruskal-Wallis with Dunn's multiple-comparisons test. (E) Contingency analyses of radiological response stratified by baseline nAb titers (detected vs not detected). ORR and DCR were computed as (CR+PR)/N and (CR+PR+SD)/N, respectively, where N denotes the number of evaluable patients. The same definitions were applied for iRECIST- and PET-derived ORR and DCR. Fisher's exact test was used to evaluate contingency between categories.

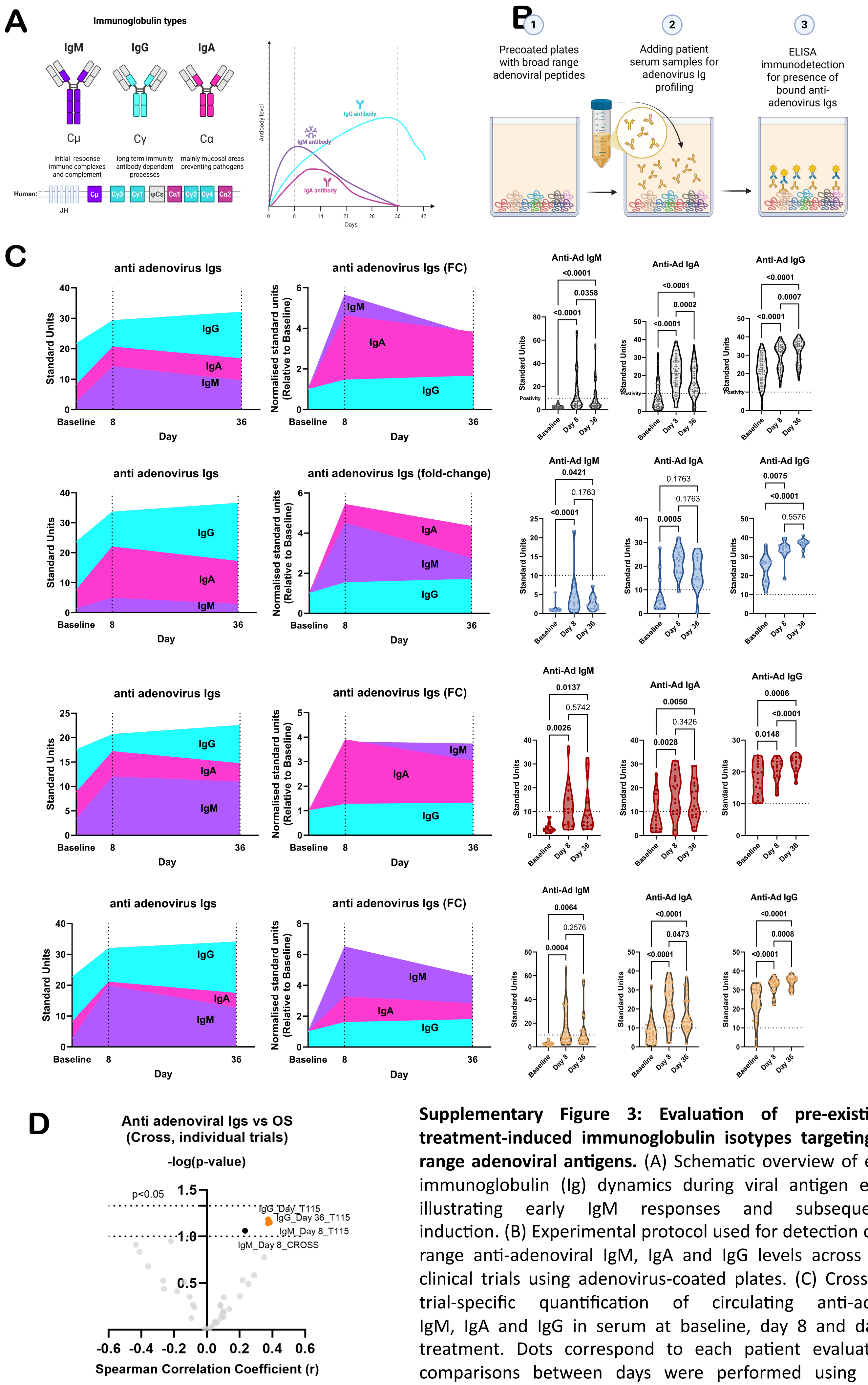

Supplementary Figure 3: Evaluation of pre-existing and treatment-induced immunoglobulin isotypes targeting broad-range adenoviral antigens. (A) Schematic overview of expected immunoglobulin (Ig) dynamics during viral antigen exposure, illustrating early IgM responses and subsequent IgG induction. (B) Experimental protocol used for detection of broad-range anti-adenoviral IgM, IgA and IgG levels across TILT-123 clinical trials using adenovirus-coated plates. (C) Crosstrial and trial-specific quantification of circulating anti-adenoviral IgM, IgA and IgG in serum at baseline, day 8 and day 36 of treatment. Dots correspond to each patient evaluated, and comparisons between days were performed using one-way ANOVA. (D) Volcano plot showing the associations between anti-adenoviral Ig levels and overall survival (OS).

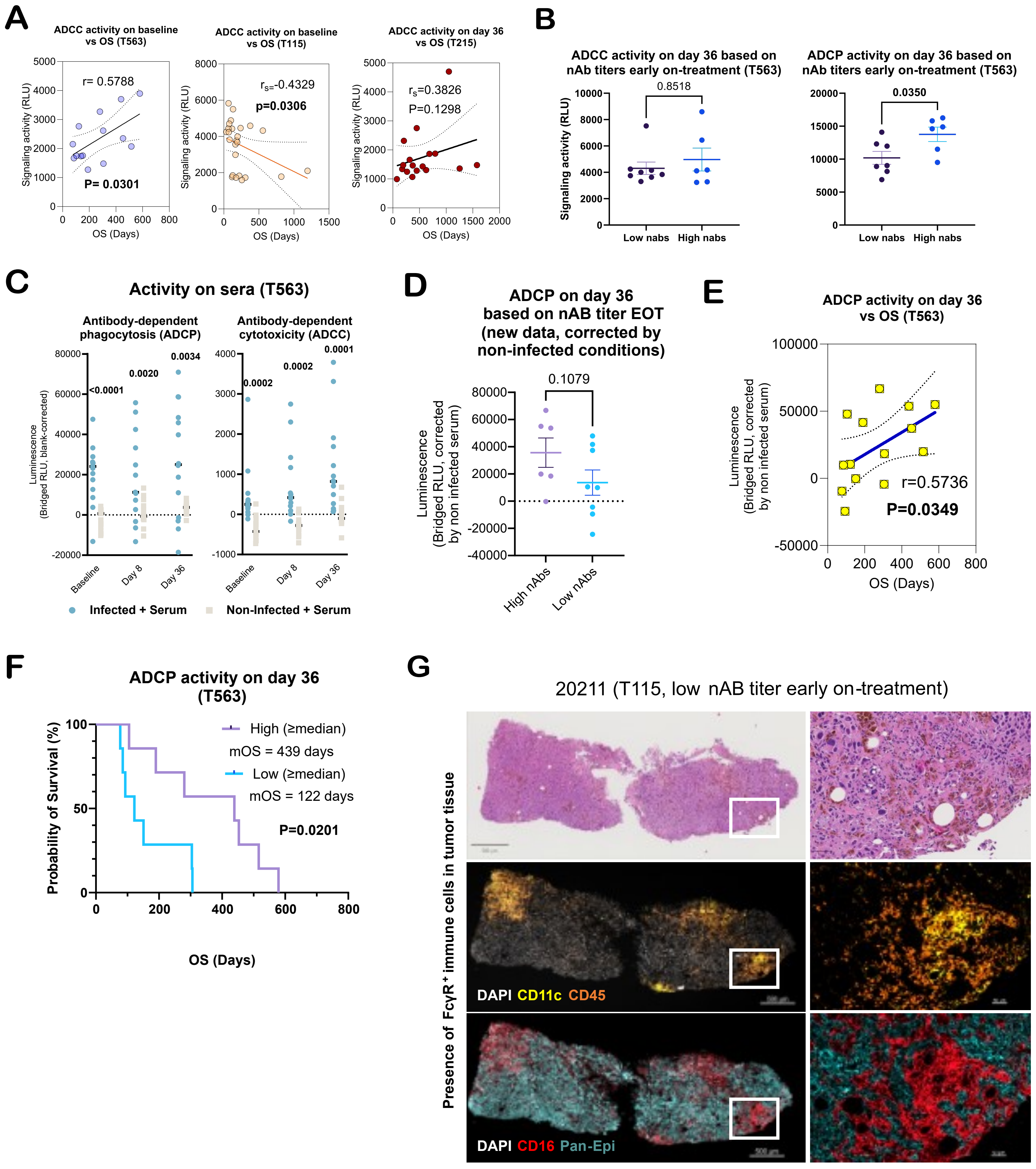

Supplementary Figure 4: Additional analyses of antibody-dependent effector functions and their relationship with antibody levels, clinical response and survival. (A) Correlation analyses between overall survival (OS) and antibody-dependent cellular cytotoxicity (ADCC) signaling activity at different timepoints, shown separately for T563, T115 and T215. Correlations were performed using Pearson/Spearman test. (B) Pairwise comparisons of ADCC and antibody-dependent cellular phagocytosis (ADCP) activity on day 36 in the T563 trial, stratified by high versus low early on-treatment nAb titers. Comparisons were evaluated using unpaired t-test. **C-F: Validation of ADCP and ADCC activity using non-infected target-cell controls in T563-PROTA**. (C) ADCP and ADCC activity measured using TILT-123-infected or non-infected A549 target cells in the presence of matched patient sera collected at baseline, day 8, and day 36. Values were corrected for the corresponding no-serum background. (D)Day 36 infection-specific ADCP activity by early-on treatment (EOT) nAb status, calculated by subtracting non-infected cells from TILT-123-infected cells after respective no-serum background corrections. For pairwise comparisons, t-tests and Wilcoxon tests were used. (E) Association between corrected day 36 ADCP activity and overall survival (OS), measured by Spearman correlation. (F) Kaplan–Meier analysis of OS according to corrected day 36 ADCP activity clustered by median, evaluated by Log-rank (Mantel-Cox) test. (G) Representative histology and mIF images from an injected tumor biopsy of a TILT-123-treated patient (20211) on day 36. H&E staining (top row) shows the overall tumor architecture and the region magnified in the inset. mIF images (middle and bottom rows) illustrate accumulation of $CD16^{+}$ immune cells in close proximity to epithelial tumor areas and spatial co-localization of $CD16^{+}$ cells with $CD11c^{+}$ infiltrates in the same regions (boxed areas). Markers are shown as follows: DAPI nuclear stain (white), CD11c (yellow), CD45 (orange), CD16 (red), pan-epithelial marker (cyan).

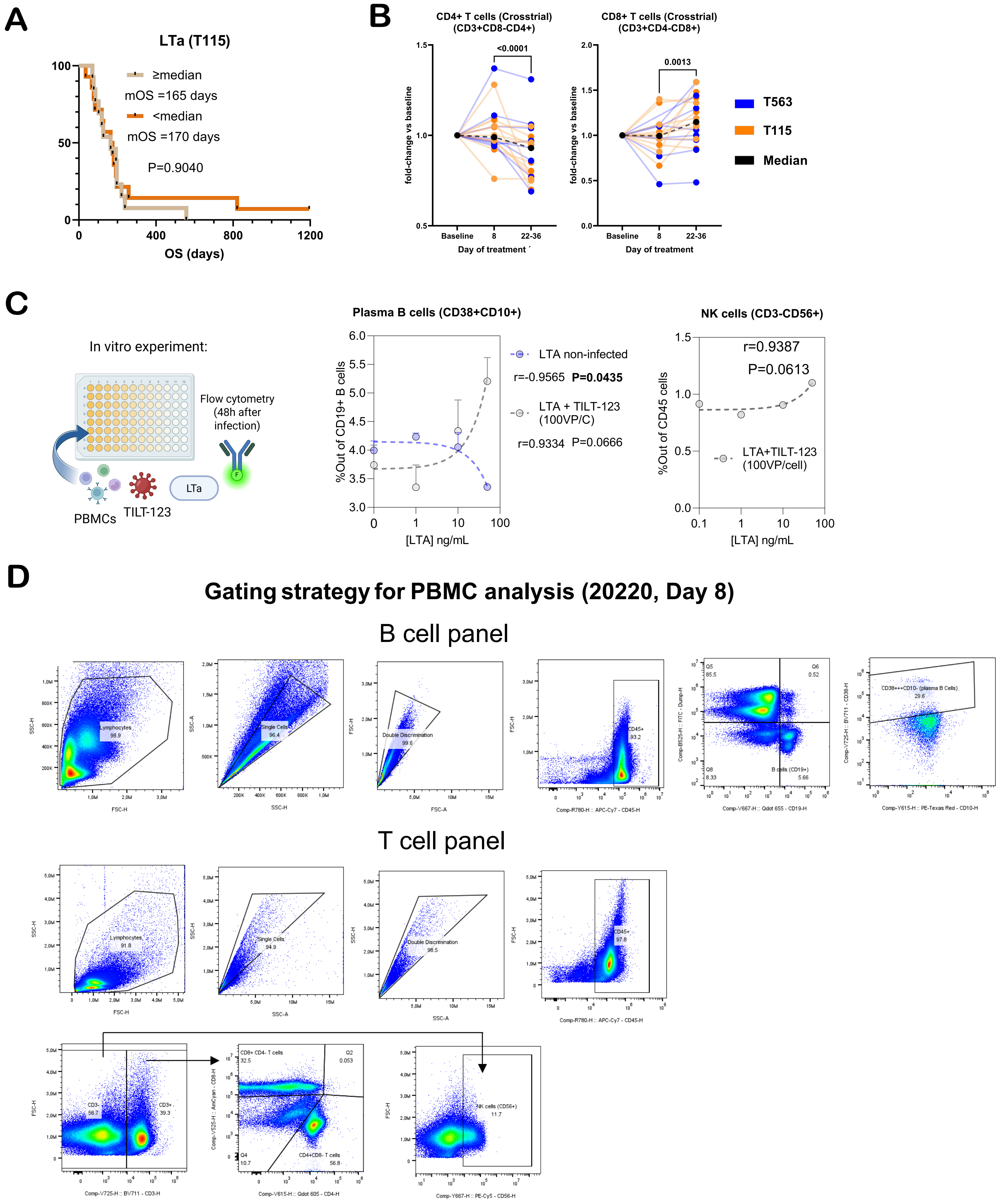

Supplementary Figure 5: Lymphotoxin-α impact on survival, T-cell dynamics and immune modulation in the context of TILT-123 treatment. (A) Kaplan-Meier survival curves for OS in the T115 trial, stratified by baseline serum lymphotoxin-α (LTa) levels (median as threshold). Survival was evaluated using Gehan-Breslow-Wilcoxon test. (B) Fold-change analysis of $CD8^{+}$ and $CD4^{+}$ T-cell populations in peripheral blood mononuclear cells (PBMCs) at baseline, day 8 and day 22/36 in selected crosstrial patients. Comparisons were evaluated using Wilcoxon test between days. (C) In vitro setup used to evaluate LTa-mediated modulation of naïve PBMCs in a dose-dependent co-culture experiment with TILT-123, and flow cytometry-based assessment of plasma B cells and NK cells as a function of LTa dose. Dose-dependent correlations were performed using Pearson test. (D) Representative gating strategy used for the identification of the main PBMC populations analyzed (B/plasma B cells, NK cells, T cells).

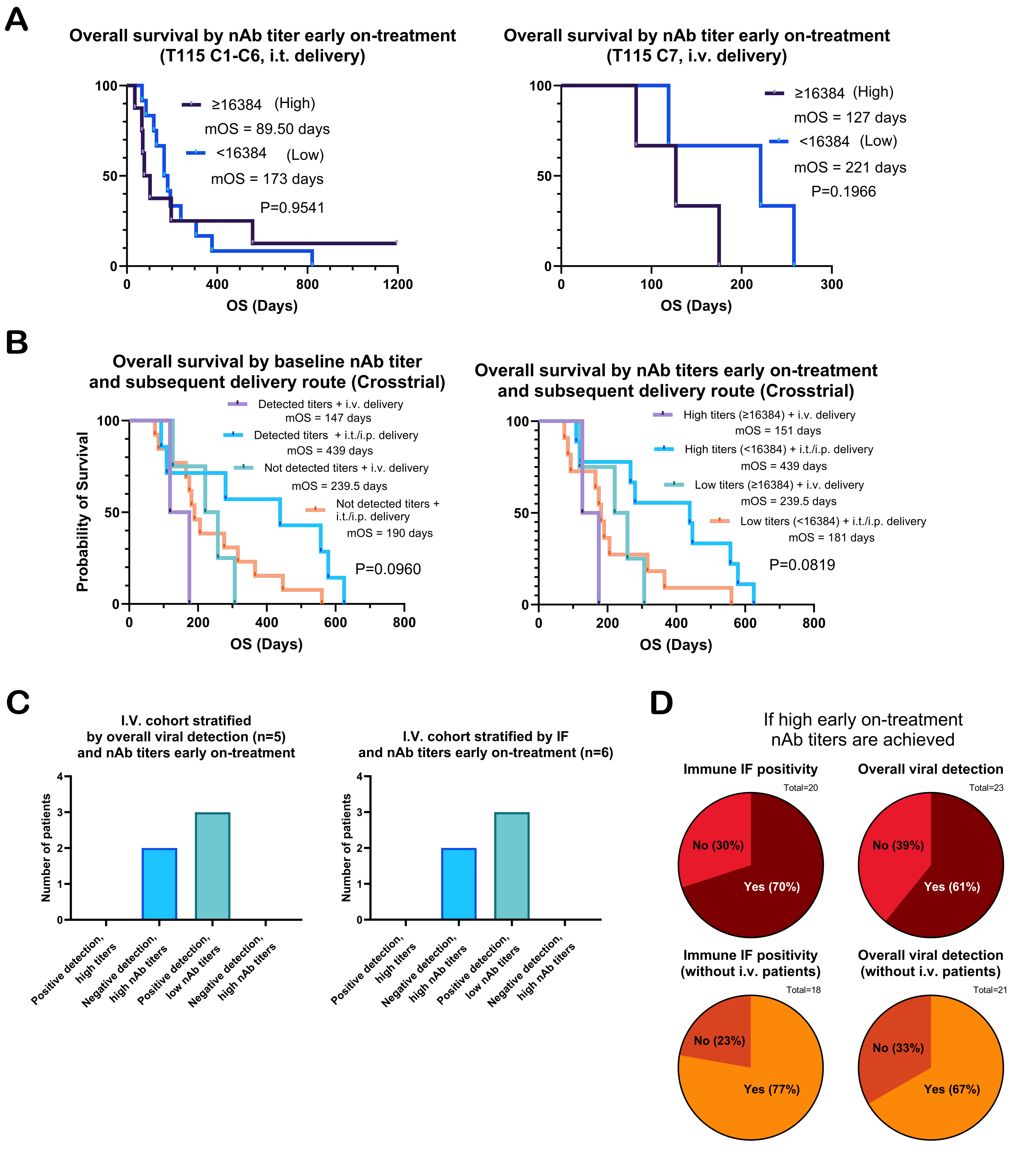

Supplementary Figure 6: Impact of intravenous versus intratumoral delivery on nAb-associated survival, viral tumor infection and immune infiltration in TILT-123-treated patients. (A) Kaplan-Meier curves for OS stratified by high versus low nAb titers achieved early on-treatment comparing T115 cohorts C1-C6 (intratumoral delivery after day 8) and C7 (fully intravenous delivery). (B) Kaplan-Meier survival curves comparing intravenous (i.v.) versus intratumoral/intraperitoneal (i.t./i.p.) administration routes in patients receiving equivalent treatment doses. Patients were stratified by baseline nAb positivity, high nAb levels on day 8 and delivery route. Log-rank test was used for all survival analyses. (C) Summary of T115 C7 patients (i.v. delivery), categorized by whether they achieved high post-treatment nAb titers and tested positive for intratumoral viral presence, as assessed by immunofluorescence (IF) and overall viral detection (qPCR and hexon staining). (D) Pie charts summarizing immune infiltration based on IF positivity and overall viral presence (qPCR, hexon staining, IF) among patients who achieved high post-treatment nAb titers, shown for the full analysis and for analyses excluding patients treated via i.v. delivery.

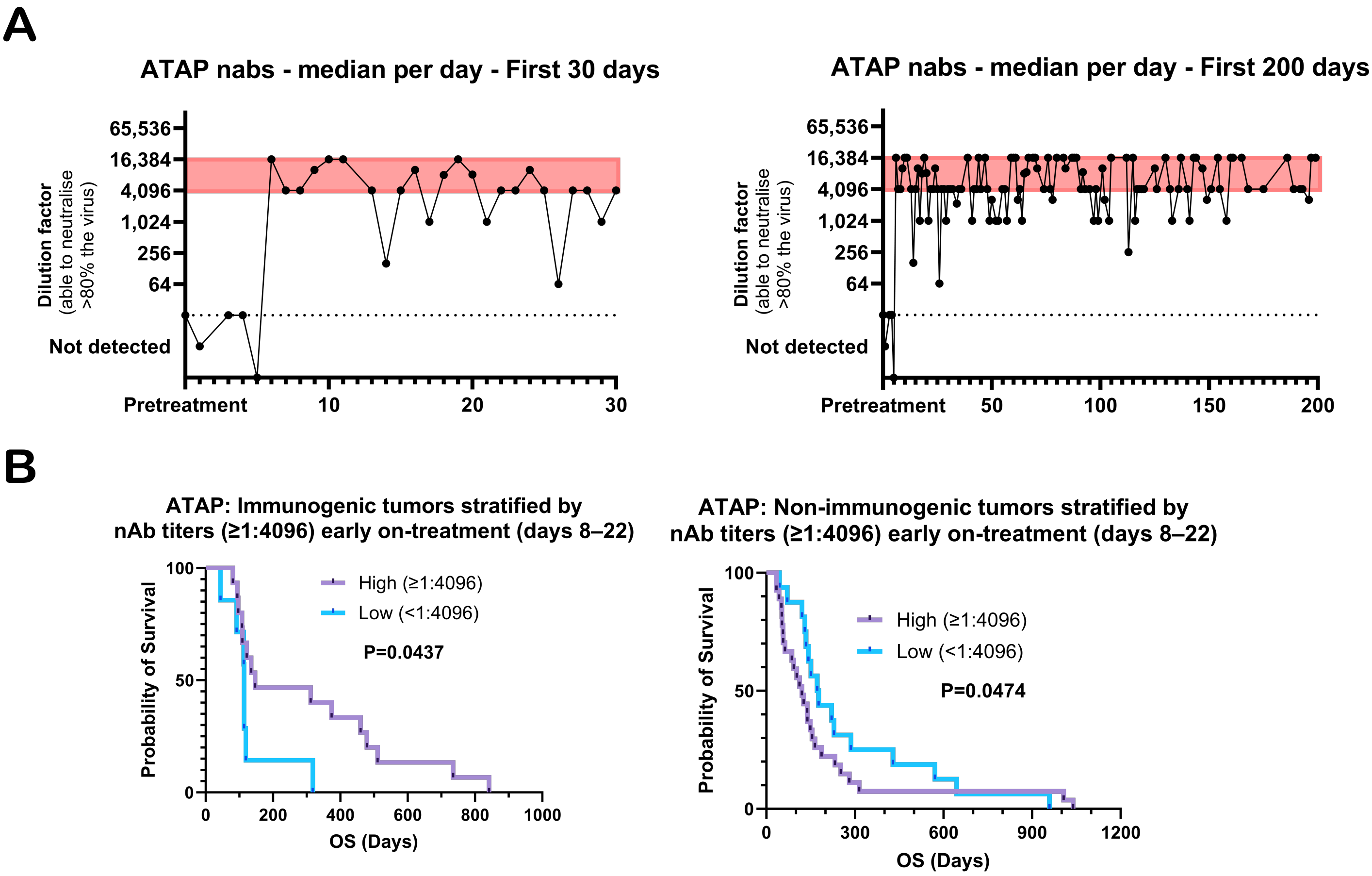

Supplementary Figure 7: ATAP validation dataset for nAb induction and tumor immunogenicity. (A) Distribution of neutralizing antibody titers in the Advanced Therapy Access Program (ATAP) dataset, indicating a median post-treatment titer of 1:4096. Line corresponds to the median value for each day evaluated. (B) Kaplan-Meier survival curves evaluating the association between nAb titers and OS in the ATAP dataset after clustering tumors by immunogenicity (higher versus lower immunogenic indications) and achievement of a titer ≥1:4096. Survival was evaluated using log-rank test.
